# Training needs and curricular priorities for managing multimorbidity in Spanish primary care: findings from a national exploratory online survey

**DOI:** 10.1186/s12875-026-03313-6

**Published:** 2026-05-06

**Authors:** Francisca Leiva-Fernández, Alba González-Hevilla, Marcos Castillo-Jimena, Aida Moreno-Juste, Juan Antonio López-Rodríguez, Paula Ara-Bardají, Isabel del Cura-González, Antonio Gimeno-Miguel, MULTIPAP Group, José María Abad-Díez, Marta Alcaraz Borrajo, Gloria Ariza Cardiel, Mercedes Aza-Pascual-Salcedo, Amaya Azcoaga Lorenzo, Ana Cristina Bandrés-Liso, Maria del Pilar Barnestein-Fonseca, María Bestué Cardiel, Kevin Bliek Bueno, María José Bujalance-Zafra, Amaia Calderon-Larrañaga, Mabel Cano del Pozo, Jonas Carmona Pirez, María Teresa Carrión de la Fuente, Mercedes Clerencia-Sierra, Jesús Díez Manglano, Jose Ángel Fuentes Ruiz, Ana Isabel González González, Francisca González Rubio, Inmaculada Guerrero Fernández de Alba, Ignatios Ioakeim Skoufa, Fernando López-Verde, Cristina M Lozano Hernández, Mónica Machón Sobrado, Alessandra Marengoni, María Isabel Márquez-Chamizo, Javier Marta-Moreno, Jesús Martín Fernández, Angel Mataix SanJuan, Inmaculada Moraga Ropero, Aida Moreno Juste, Christiane Muth, Victoria Pico-Soler, Beatriz Poblador-Plou, Alexandra Prados-Torres, Juan Daniel Prados-Torres, Elena Polentinos Castro, Francisca Rius Díaz, Ricardo Rodríguez Barrientos, José María Ruiz-San-Basilio, Mercedes Rumayor Zarzuelo, Luis Sánchez Perruca, Raquel Sánchez Ruano, Teresa Sanz Cuesta, Maria Eugenia Tello Bernabé, José María Valderas Martínez, Francisco Javier Orellana Lozano, Jesús Sepúlveda Muñoz, Rafael Sánchez Jordán, Amparo Escobar Pérez, Javier Martín Izquierdo, Macarena Toro Sainz, Concepción Rodríguez García, Trinidad Peñuela Ruiz, José Antonio Navarro Martín, María Rosario Rodríguez Rivera, Yolanda Aguilar Heredia, Antonio Ignacio Martínez Sarmiento, Beatriz Pascual de la Pisa, María José García Lozano, Alejandro García Carrera, Noelia Juan Tordesillas, María José Fernández Jiménez, José Manuel Navarro Jiménez, María Carmen Ruiz Ciudad, Rubén Luciano Vázquez Alarcón, María Isabel Navarro Gallego, Leovigildo Ginel Mendoza, Luz Pilar de la Mota Ybancos, Jaime Sasporte Genafo, María José Alcaide Rodríguez, Elena Barceló Garach, Beatriz Caffarena de Arteaga, María Dolores Gallego Parrilla, Catalina Sánchez Morales, Elisa María Alcantarilla Reyes, Marta Álvarez de Cienfuegos Hernández, Nuria Segura Domínguez, Maria del Mar Loubet Chasco, Irene Martínez Ríos, Elena Mateo Delgado, Laura Orellana Martín, María Dolores Merino Moyano, María Cristina Moral Merchán, Esther Martín Aurioles, María Inmaculada Rodríguez González, Sylvia Hazañas Ruiz, María Auxiliadora Nieves Muñoz Escalante, Eva Noelia Gallego Castillo, Esperanza Mora García, Enrique Leonés Salido, Maria Antonia Máximo Torres, Maria Luisa Moya Rodríguez, María Encarnación Peláez Gálvez, José Manuel Ramírez Torres, Cristóbal Trillo Fernández, Estefanía Cámara Sola, Sergio Fons Cañizares, María Paz Ortigosa Arrabal, Teresa Quesada Fernández, Silvia Rodríguez Moreno, Maria Dolores García Martínez Cañavate, Maria del Mar Gil Mellado, Maria Victoria Muñoz Pradilla, Ana Sánchez Silvestre, María Jesús Torrubia Fernández, María José González Vega, María Victoria Almagro Martín-Lomeña, Caridad Serrano González, José Leiva Fernández, Virginia Castillo Romero, Maria José Clavijo Peña, Rubén Vázquez Alarcón, Rafael Ángel Maqueda, Gloria Aycart Valdés, Ana Maria Fernández Vargas, Irene García, Antonia González Rodríguez, Maria Carmen Molina Mendaño, Juana Morales Naranjo, Francisco Serrano Guerra, Gabriel Francisco Narbona Carrión, Hervé Michel Bertevas, Ascensión Saura Campos, María Begoña Abadía-Taira, Eugenio Galve Royo, José Fernando Tomás Gutiérrez, Carmen Sánchez Celaya del Pozo, Ana Carmen Giménez-Baratech, Lara Sanz-Burgos, Mercedes Abad-Royo, Teodoro Corrales Sánchez, Eustaquio Dendarieta Lucas, Carmen Camats-Franco, José Manuel Cortés-Pellicer, Paula Herrero-Solsona, Miguel Guiu-Campos, Nima Peyman-Fard-Shafi-Tabatabaei, José Ignacio Torrente Garrido, Concepción García Aranda, Marina Pinilla Lafuente, Ma. Teresa Delgado Marroquín, Mercedes López-Echevarría, Maria Teresa Martín Nasarre de Letosa, Elena Gascón del Prim, Noelia Sorinas Delgado, Maria Rosario Sanjuan Cortés, Fernando Barrera-Linares, Sandro Daniel Carrillo-Soria, Ana Belén Esteban-Gimeno, Beatriz López-Alonso, Anabel Hernández-Bono, Enrique Martínez-Ayala, Adriana Martínez-Manero, Raquel Martínez-Sánchez, Yolanda Naya-Mateu, María Lourdes Clemente-Jiménez, María Paz Leon-Martínez, Liliana Bilbie Lupchian, Mª Rosario Sanjuan-Cortés, Elisa Pilar Salazar-González, Mª Elena Charte-Alegre, Mª Jesús Mur-Lazuela, Mónica Pascual-Franco, Mª José Gracia Molina, Javier Cuartero Bernal, Mª Victoria Asín Martín, Susana García Domínguez, Pilar Arizon-Deza, Carmen García-Gutiérrez-Muñoz, Teresa García-Ruiz, Gloria Navarro-Aznárez, José Porta Quintana, Valentina Martín Miguel, Esther Mateo de las Heras, Carmen Esteban Algora, Carlos Alcober-Pérez, María Paz Navarro-Tausiet, Mª Elena Lacasa-Serrano, Ana Cristina Maza-Invernón, Jaime Peleato-Sánchez, Adolfo Cajal Marzal, José Miguel Buñuel-Granados, Ainara Alonso-Valbuena, Mónica Lasheras-Barrio, Isabel Ibarrondo-Fernández-Ladreda, Mª Rosa López-Aylon, María José Rodríguez-Fabre, Isabel Rubio-Gutiérrez, Selma Valverde-Aranda, Antonio Luis Oto Negre, David Santos Muñoz, María Elisa Viñuela Benítez, Estrella Gutiérrez Ocana, Raquel García Ocaña, M Ángeles Miguel Abanto, Francisca García De Blas González, Eduardo Díaz García, Juan Carlos García Álvarez, Cristina Guisado Pérez, Alberto López García Franco, Sonia Redondo De Pedro, Ana Ballarín González, Maria Isabel Ferrer Zapata, Esther Gómez Suarez, Fernanda Morales Ortiz, Lourdes Carolina Peláez Laguno, José Luis Quintana Gómez, Enrique Revilla Pascual, Carlos Fernando González García, Yolanda Beatriz Sánchez Fernández, Yolanda Fernández Fernández, Blanca Gutiérrez Teira, Esther Barrio Higelmo, Eva María Rioja Delgado, Irina Lopez Larrayoz, María Luz Seara Lozano, Julio Cesar Fernández Sánchez, María Teresa San Miguel Marinero, María Jesus Fidalgo Baz, Sara Ares Blanco, Jorge Ignacio Gómez Ciriano, José Damián Garcés Ranz, Laura Santos Franco, María Celeste García Galeano, Sara Morcillo Cebolla, Tomás Rossignoli Fernández, Francisco Ramón Abellán López, Carlos Casado Álvaro, Paulino Cubero González, Santiago Manuel Machín Hamalainen, Raquel Mateo Fernández, Cesar Sánchez Arce, Jorge Olmedo Galindo, Marta Pinel González, Rosa María Redondo Romero, Adnaloy Helena Estrada Leon, Blanca Sanz Pozo, Luis Enrique Morales Cobos, María Del Prado Garcia Garcia-Alcañiz, Marisol Lorenzo Borda, Claudia López Marcos, Soledad Lorenzo Borda, Juan Carlos Moreno Fernández, Belén Muñoz Gómez, Enrique Rodríguez De Mingo, Vera González García, María Del Pilar Muñoz Molina, Yasmin Drak Hernández, Alejandro Rabanal Basalo, Ana María Abad Esteban, María De Los Ángeles Rollan Hernández, Mónica Fuster Tozer, Raquel Carretero Ramos, Rebeca Mielgo Salvador, Carmen María Muros Muñoz, María Cristina Cáceres Cortés, María Pastor Estebanez, Mercedes Fernández Girón, Juan Pedro Calvo Pascual, Maria Paloma Morso Peláez, Julio Sánchez Salvador, Jeannet Dolores Sánchez Yépez, Ana Sosa Alonso, Antonia De Colosia Zuil, Esteban Pérez Gutiérrez, Isabel Tejero García, Jaime Innerarity Martínez, Margarita Gómez Barroso, María Del Mar Escobar Gallegos, María Jesus Bedoya Frutos, Marta Inmaculada Del Olmo Ribagorda, Petra María Cortés Durán, Pilar Tardáguila Lobato, Raquel Yolanda Terrón Barbosa, Maria del Mar Álvarez Villalba, Antonio Ramos Blanco, Aránzazu López Villalvilla, Beatriz Cinta Bella, Cristian Varela Varela, Francisca Garcia Rodriguez, Gema María Saiz Ladera, Guillermina López Fernández, Lourdes Orozco Barrenechea, María Begoña Zafra De Gea, Nuria García Arpa, Tamara Ewa Barnas, Ana Isabel Carbonero Martín, María José Rojas Giraldo, Purificación Magán Tapia, María Angélica Fajardo Alcántara, Maria Canto De Hoyos Alonso, Maria Aránzazu Murciano Antón, Manuel Antonio Alonso Pérez, Ricardo De Felipe Medina, Amaya Nuria López Laguna, Eva Martínez Cid De Rivera, Iliana Serrano Flores, Maria Jesús Sousa Rodríguez, Maria Soledad Núñez Isabel, Jesús Mª Redondo Sánchez, Pedro Sánchez Llanos, Lourdes Visedo Campillo, Alberto Cotillas Rodero, Beatriz López Serrano, María Del Carmen Del Rodriguez Fernández, Carmelina Sanz Velasco, Jose Ignacio Aza Pascual-Salcedo, Carolina Lopez Olmeda, Milagros Rico Blázquez, Mª Gloria Ariza Cardiel, Angel Mataix SanJuan, Mariel Morey Montalvo, Mercedes Rumayor Zarzuelo, Rafael Rotaeche del Campo, Ana Toledo Chávarri, Amado Rivero Santana, Lidia García Pérez, Beatriz León Salas, Ana María Pascual y Medina, Alejandro Pérez Guerra, José Ramón Vázquez Díaz, Lidia Esther Nuez de la Era, Juana de la Cruz González Gonzáles, Pedro Jorge Araujo, Manuel Enrique Méndez Abad, Janet Delgado, Beatriz Rodríguez Martín, Yolanda Álvarez Pérez, Andrea Duarte

**Affiliations:** 1 Instituto de Investigación Biomédica de Málaga y Plataforma en Nanomedicina–IBIMA Plataforma Bionand, Málaga, Spain; 2 Network for Research On Chronicity, Primary Care, and Health Promotion (RICAPPS), Institute of Health Carlos III (ISCIII), Madrid, Spain; 3 Multiprofessional Teaching Unit of Community and Family Care Primary Care District Málaga-Guadalhorce Knowledge Management Unit Málaga-Guadalhorce Health District, Andalusian Health Services, Málaga, Spain; 4 Department of Pharmacology and Paediatrics, Faculty of Medicine, University of Malaga, Andalucía Tech, PhD Program in Biomedicine, Translational Research and New Health Technologies, Málaga, Spain; 5 UGC (Clinical Management Unit) Alozaina, Health District Valle del Guadalhorce, Málaga, Spain; 6 Primary Care Health Centre Campillos, Northern Málaga Integrated Healthcare Area, Andalusian Health Service, Campillos, Málaga, Spain; 7 EpiChron Research Group, Aragon Health Sciences Institute (IACS), IIS Aragón, Miguel Servet University Hospital, Zaragoza, Spain; 8 San Pablo Healthcare Centre, Aragon Health Service (SALUD), Zaragoza, Spain; 9 Ricardos General Health Center (Centro de Salud General Ricardos), Madrid, Spain; 10 Department of Medical Specialties and Public Health, School of Health Sciences, Rey Juan Carlos University (Universidad Rey Juan Carlos), Madrid, Spain; 11 Group 34: Multimorbidity, Chronic Diseases and Health Services. Primary Care. Instituto de Investigación Sanitaria Gregorio Marañon IISGM, Madrid, Spain; 12 Research Unit, Primary Health Care Management Madrid Health Service. Madrid, Madrid, Spain; 13 Ageing Research Center, Karolinska Institutet and Stockholm University, Stockholm, Sweden

**Keywords:** Multimorbidity, Primary Health Care, Education, Curriculum, Surveys and Questionnaires

## Abstract

**Background:**

Multimorbidity is highly prevalent and poses clinical and organisational challenges, yet the training of doctors and nurses continues to focus mainly on single conditions. In recent years, training interventions for healthcare professionals on multimorbidity have emerged, but evidence of their effectiveness remains limited. Understanding the learning needs of professionals is essential for guiding educational programmes.

This study aimed to identify the perceived training needs of primary care physicians and nurses in the management of multimorbidity and to contribute to a proposal of updated curriculum content.

**Methods:**

Exploratory cross-sectional study using an online survey of doctors and nurses in primary care and out-of-hospital emergency services in Spain. The questionnaire was developed based on the literature, existing curriculum frameworks and previous findings from the MULTIPAP study, and collected sociodemographic, professional and educational data, as well as perceptions about resources, difficulties and training needs in multimorbidity. Descriptive, bivariate, and multivariable analyses were conducted, and open-ended responses were analysed using thematic content analysis.

**Results:**

The main difficulties and training needs concerned medicines management and diagnostic and therapeutic challenges, followed by communication and time management. Participants valued patient–caregiver-centred care, medicines management, and care coordination. An online format with videos was preferred. Differences were observed by profession, gender, field of work, trainee status, and prior participation in the MULTIPAP study. The proposed curriculum comprised eight competency groups, including professionalism and social sciences along with other key issues, especially clinical, communication, and management skills.

**Conclusion:**

Professionals share many training needs, obstacles, and resources related to multimorbidity care, with some differences between subgroups. These findings informed a proposal for an updated curriculum content.

**Supplementary Information:**

The online version contains supplementary material available at 10.1186/s12875-026-03313-6.

## Background

The coexistence of multiple long-term conditions in an individual or multimorbidity represents an emerging public health issue due to its impact and increasing prevalence worldwide [1]. According to a recent meta-analysis, approximately 37% of the world’s adult population live with multimorbidity [2]. This percentage doubles among those aged over 65 years and exceeds 80% in individuals in the last decades of life [3] with similar figures observed in Spain [4, 5]. Nonetheless, these prevalence rates vary considerably depending on the definitions and data sources used [6–8].

Multimorbidity has significant adverse implications for individuals, healthcare systems, and societies. People living with multimorbidity experience deteriorating physical, psychological, social, and sexual health [9], increased disability, polypharmacy, and poorer quality of life [10–12]. Multimorbidity is also linked to increases in costs and use of healthcare services, including avoidable hospitalisations [13, 14].

Primary care (PC) is the central framework for supporting patients with multimorbidity, given its comprehensive, longitudinal, and community-based approach [15, 16]. PC professionals usually manage multiple health problems simultaneously, coordinating and adapting care to each individual [15, 17]. The Ariadne Principles proposed by Muth et al. [18] have helped to conceptualise this patient-centred approach to multimorbidity management. However, training of medical and nursing students remains focused on specific pathologies, limiting their ability to manage complex cases [15, 19].

In Spain, where PC is the cornerstone of multimorbidity management within the National Health System, research on this topic has grown through initiatives such as the Research Network on Chronicity, Primary Care and Health Promotion (RICAPPS) [20]. However, coverage of multimorbidity in undergraduate curricula remains limited, and the explicit incorporation of multimorbidity into formal postgraduate training has only recently begun. The prior Family and Community Medicine programme approved in 2005 [21] was largely focused on single-disease approaches and specialty-based rotations, while the Family and Community Nursing programme [22], although it contemplates continuity of care, community orientation, and chronic disease management, does not define multimorbidity as a core competency. The new Family and Community Medicine programme [23] introduces a competency-based framework that focus on multimorbidity, frailty, polypharmacy, and care coordination. Despite this update, most practising and training professionals were educated under earlier curricula, and little is known about their perceived educational needs for managing multimorbidity.

Several recent interventions to improve care for patients with multimorbidity have been published [24–32], some including professional training activities. However, the evidence of their effectiveness is inconclusive [33]. Advances in this field depend on a greater understanding of the experiences and perceived learning needs of PC professionals involved in managing multimorbidity, particularly in healthcare systems where formal training has traditionally focused on single-disease models. The systematic review by Lewis et al. [25] provides an essential foundation, suggesting that healthcare professionals would benefit from curricular content in areas such as clinical skills, communication, teamwork, information technology, critical evaluation, and management skills, and highlighting the need to identify training formats with the greatest acceptance and clinical impact [25]. Educational planning based on perceived needs allows us to address real challenges and promotes more meaningful learning that can be integrated into practice [34, 35].

Given the recent evolution of training programmes and the lack of evidence on how well current healthcare professionals feel prepared to manage multimorbidity, this study aimed to identify perceived training needs in multimorbidity care among PC professionals and to explore differences between subgroups, including professional and sociodemographic characteristics. The outcomes of the competencies and skills identified by the participants in this study, together with those proposed by Lewis et al. [25], will be integrated to propose an updated curriculum on multimorbidity.

## Methods

We conducted an exploratory, observational, descriptive, and cross-sectional study based on an online survey within the framework of the MULTIPAP study and its associated clinical trials (MULTIPAP [26] and MULTIPAP Plus [36]), complementing the eMULTIPAP training intervention [28]. Following the CHERRIES checklist [37, 38], cookies prevented multiple entries, and informed consent was obtained as the first mandatory question, with full details provided in the participant information sheet. See Table AF1 in the additional files. The study protocol has been previously published [39].

### Participants, sampling, and data collection

The target population comprised physicians and nurses from health centres and out-of-hospital emergency rooms of the Spanish National Health System. A non-probability convenience sampling strategy was used, combining purposive and snowball sampling through digital means (WhatsApp messages sent to specific healthcare professionals and general emails to health districts, teaching units, and scientific societies). Accordingly, findings are interpreted as exploratory rather than nationally representative.

Sample size was calculated to estimate proportions related to the primary objective of describing perceived training needs in multimorbidity among PC professionals. In the absence of prior estimates for the expected proportions, maximum uncertainty was assumed (*p* = 0.5), which provides the most conservative sample size estimate. Considering a 95% confidence level and a precision of 5%, the sample size was 384 participants. Sample size was also estimated for Likert-type items (95% confidence level, 4-point Likert scale), resulting in a minimum requirement of 104 participants. As this value was lower than the sample size required for the estimation of proportions, the larger sample size (*n* = 384) was chosen.

Sequential invitations were sent between November 2021 and July 2022 until the target sample size was reached.

### Questionnaire and variables

An anonymous questionnaire was specifically developed for this study, informed by the literature and by previous findings from the MULTIPAP study [26, 36], as well as by previously described competencies for multimorbidity management in PC [25]. The preliminary version was pilot-tested between July and August 2021 with three PC physicians and two nurses to assess clarity, relevance, feasibility, and completion time. The mean reported completion time was approximately 15 min. Feedback from pilot participants was collected and discussed within the research team, and minor modifications were introduced, including rephrasing certain questions, correcting response options, clarifying Likert-scale anchors, and adjusting the order and format of some items.

Because the questionnaire was designed as an exploratory instrument to identify perceived training needs rather than to measure latent constructs or to develop a new measurement tool, no formal psychometric validation was performed subsequently. The questionnaire consisted of several sections combining descriptive categorical items with open-ended questions intended to capture professionals’ experiences, perceived gaps, and educational expectations.

The survey was created using LimeSurvey® and was structured into four sections: (i) informed consent; (ii) sociodemographic, professional, and educational information; (iii) competency assessment using Likert-scale items; and (iv) open comments. It covered four main areas: (1) sociodemographic and professional characteristics; (2) training in multimorbidity; (3) perceptions, challenges, and training needs related to multimorbidity care; and (4) conceptual understanding and perceived importance of competencies associated with multimorbidity management. The survey included 14 closed questions and six open-ended questions, four of which collected free-text responses on perceived training needs, short-term learning interests, main care difficulties, and key tools to address them. In addition, 12 statements based on the curriculum proposed by Lewis et al. [25] were used to assess competencies relevant to multimorbidity care. Each statement was rated on a predefined 4-point Likert scale ranging from “none” to “maximum importance”. The full English version of the questionnaire (AF2a) and a summary of the survey variables (Table AF2b) are provided as additional files.

### Data processing and analysis

Descriptive statistics were calculated: quantitative variables were expressed as measures of central tendency and dispersion; qualitative variables as absolute and relative frequencies; and Likert responses as medians and frequencies. Bivariate analyses were performed to examine training needs, priorities, tools, and perceived difficulties according to age, gender, profession, work setting, trainee status, previous participation in the MULTIPAP study, and the operational definition of multimorbidity selected by respondents. Student’s t-test, Pearson’s chi-squared test, and Fisher’s exact test were used as appropriate depending on the type and distribution of the variables. In addition, exploratory multivariable binary logistic regression analyses were performed for selected outcomes to assess whether observed associations remained after adjustment for potential confounders. Selected dichotomous outcomes were used as dependent variables, separately. Independent variables included profession, gender, work setting, trainee status, and participation in the MULTIPAP study, and were entered simultaneously into the models. Adjusted odds ratios (aOR) with 95% confidence intervals (CI) were calculated. Given the exploratory design and the use of convenience sampling, these analyses were considered hypothesis-generating. Statistical significance was p < 0.05. All statistical analyses were performed using IBM SPSS Statistics version 22.0.

Open-ended question data were analysed using thematic content analysis, integrating both inductive and deductive approaches. Two researchers (FLF and AGH) independently reviewed all responses multiple times to familiarise themselves with the material, then segmented the text into meaning units for coding. Initial codes were generated inductively, without a predefined framework, and each researcher independently applied them across all responses to the four open-ended questions. For the qualitative analysis, every available response was considered. Incomplete questionnaires were excluded from quantitative analyses but were included in the qualitative dataset if they contained at least one interpretable response relevant to the study objectives. Non-informative entries (e.g., “don’t know”, “.”, “-”, or otherwise irrelevant) were excluded. As a result, the number of responses analysed varied by question (Training needs: n = 399; Training priorities: n = 386; Difficulties: n = 402; Tools: n = 383). Coding proceeded iteratively until no substantially new codes emerged across the dataset.

The two researchers subsequently compared their coding and developed a shared codebook through discussion, including a table of equivalences aligning code labels and definitions (Supplementary Tables AF3–AF4). Discrepancies were resolved through consensus to minimise individual interpretative bias. The organisation and comparison of codes were supported using a structured spreadsheet developed ad hoc in Microsoft Excel (no computer-assisted qualitative data analysis software was used). The spreadsheet allowed systematic comparison of codings and tracking of code revisions across the analysis.

**Fig. 1:**
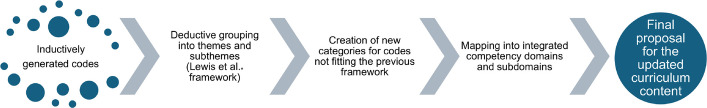
Mapping process from qualitative analysis to the proposed curriculum based on eight competencies

In a second analytical step, the inductively generated codes were grouped, where appropriate, into the competency themes and subthemes described by Lewis et al. [25], which were used as a deductive framework to organise the data. Codes that did not fit the existing framework led to the creation of new categories. To ensure analytical consistency and conceptual traceability, a glossary of operational definitions with illustrative examples was developed and used throughout the coding process (Supplementary Table AF5). The full list of themes and subthemes corresponding to the deductive framework described by Lewis et al. [25], along with the inductively generated codes, is provided in Supplementary Table AF6. Finally, the updated curriculum proposal was informed by this qualitative analysis, mapping the inductive codes onto the selected framework to identify key domains and learning objectives relevant to PC practice (Fig. 1).

Both researchers were involved in the MULTIPAP study and have clinical experience in multimorbidity in PC, providing contextual familiarity with the study topic. To mitigate potential interpretative bias related to this positionality, independent coding, comparison of codes, and consensus procedures were applied. Responses were analysed in Spanish with minimal normalisation (spelling correction or unification of equivalent terms) without altering the original meaning.

## Results

**Fig. 2:**
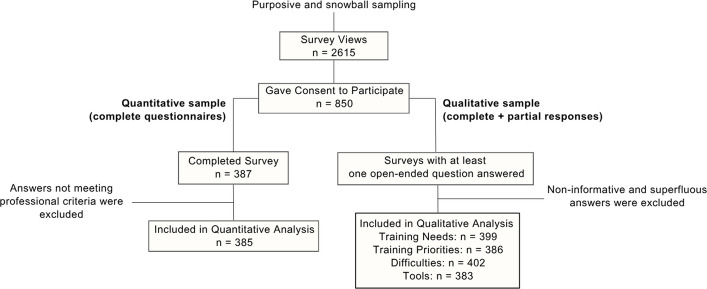
Sampling and data processing flow chart

The survey included 2615 individual views. A total of 385 completed questionnaires were included in the quantitative analysis. In addition, open-ended responses from incomplete questionnaires were included in the qualitative analysis, resulting in varying numbers of responses analysed for each question (see Fig. 2). Table 1 presents the main characteristics of participants in the survey. The mean age of participants was 45.9 years (standard deviation 12), 280 (72.7%) were women, 270 (70.2%) were physicians, and 227 (59%) worked exclusively in urban settings. The distribution of professions, training status, and professional setting in the study sample was compared with national PC workforce data from the Spanish Ministry of Health (Supplementary Table AF7). Geographically, responses were obtained from 45 of the 52 Spanish provinces, with the highest contributions from Málaga (19%), Madrid (7.5%), Cantabria (7%), Burgos (5.5%), and Barcelona (4.9%), while participation was absent in Ávila, Castellón, Córdoba, Guadalajara, Lugo, Tarragona, and Ceuta. Additional sample characteristics with full provincial and regional distributions are reported in Supplementary Tables AF8–AF9.

**Table 1:** Sample characteristics of survey participants (n = 385), including distribution by profession (physicians, nurses, and residents)

Characteristic	n (%) or mean (SD)
Age	45.9 (12.0)
Gender	
Male	104 (27.0)
Female	280 (72.7)
Other	1 (0.3)
Survey distribution channel	
Corporate email	138 (35.8)
Personal email	72 (18.7)
Social media	81 (21.0)
Instant messaging	94 (24.4)
Profession	
Doctor	254 (66.0)
Nurse	99 (25.7)
Family and Community Medicine resident	16 (4.2)
Family and Community Nursing resident	16 (4.2)
Current professional role	
Primary care Team	284 (73.8)
Out-of-hours/emergency services	21 (5.5)
Both	61 (15.8)
Other	19 (4.9)
Work setting	
Urban	227 (59.0)
Rural	113 (29.4)
Both	43 (11.2)
Other	2 (0.5)
Participation in MULTIPAP	
Yes	37 (9.6)
No	348 (90.4)
Previous training in multimorbidity	
Yes	274 (71.2)
No	111 (28.8)

*SD* standard deviation, MULTIPAP Spanish research project on multimorbidity in primary care

Recruitment was nationwide and non‑probability; interpretative considerations are detailed in Methods and Limitations.

### Definition of multimorbidity

The most selected definition of multimorbidity was “*The presence of a chronic disease in combination with at least one additional disease (acute or chronic), biopsychosocial factor, or somatic risk factor within the same individual*” [40] chosen by 52.7% of respondents. This was followed by “*The coexistence of two or more chronic health conditions in the same individual*” [15] (26.5%). Fewer participants (20%) reported using the operational threshold for complex multimorbidity found in some publications, “*Coexistence of three or more chronic health conditions in the same individual*” [41].

When responses were compared according to the operational definition of multimorbidity used by participants, most perceived training needs, difficulties, priorities and tools were similar across groups. A statistically significant difference was observed only for the prioritisation of clinical skills (p = 0.014), which was more frequently reported by respondents using the WHO or ≥ 3 conditions definitions (Table AF10).

### Evaluation of predefined competencies

**Fig. 3:**
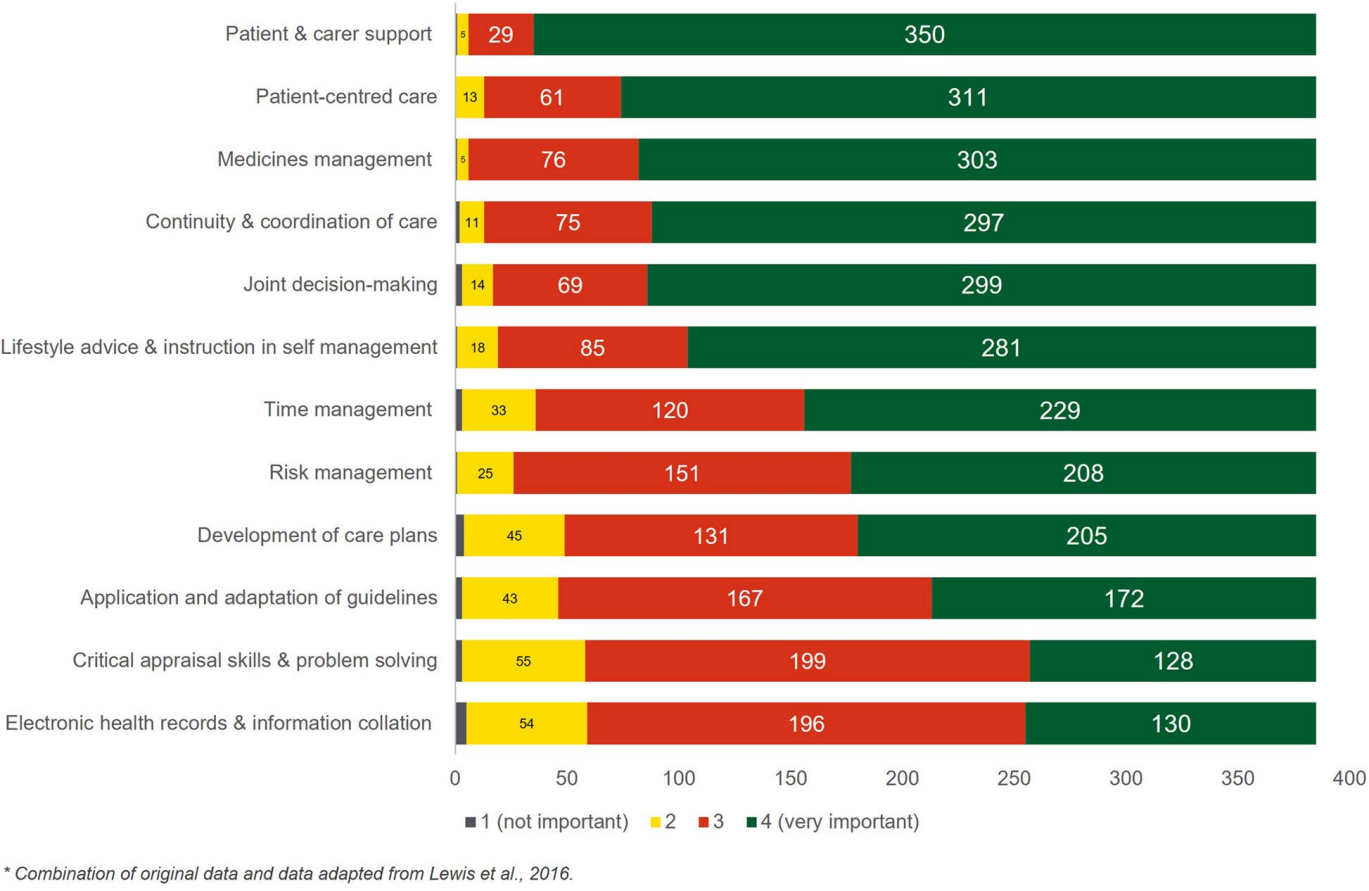
Evaluation of the 12 predefined curricular competencies

Most participants assigned high importance to the 12 competencies adapted from the curriculum proposed by Lewis et al*.* [25], highlighting support for patients and caregivers (90.9%), patient-centred care (80.8%), and medicines management (78.7%), as shown in Fig. 3 and Supplementary Table AF11.

### Difficulties, tools, training needs, and priorities

Responses to the open-ended questions about difficulties, tools, training needs and training priorities were organised into eight themes and 18 subthemes based on 194 inductive codes (Fig. 1 and Supplementary Table AF6). Two new categories were added to the classification proposed by Lewis et al*.*: [25] professionalism and social sciences.

**Fig. 4:**
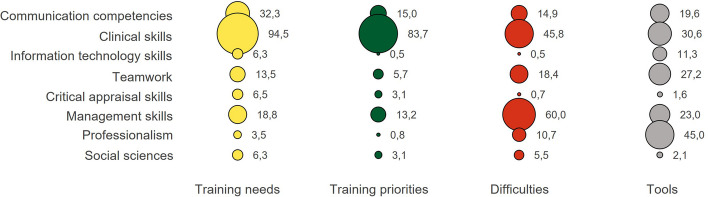
Difficulties, tools, training needs and priorities in caring for patients with multimorbidity (%)

The main difficulties identified were management skills (60%) and clinical skills (45.8%). The main tool recognized was professionalism (45%) followed by clinical skills (30.6%) and teamwork (27.2%). There was notable overlap between training needs and priorities within the category of clinical skills (94.5%), especially in medicines management and diagnostic and therapeutic challenges, followed by communication competences (32.3%) and management skills (18.8%). These findings are illustrated in Fig. 4 and detailed in Supplementary Table AF12.

**Fig. 5:**
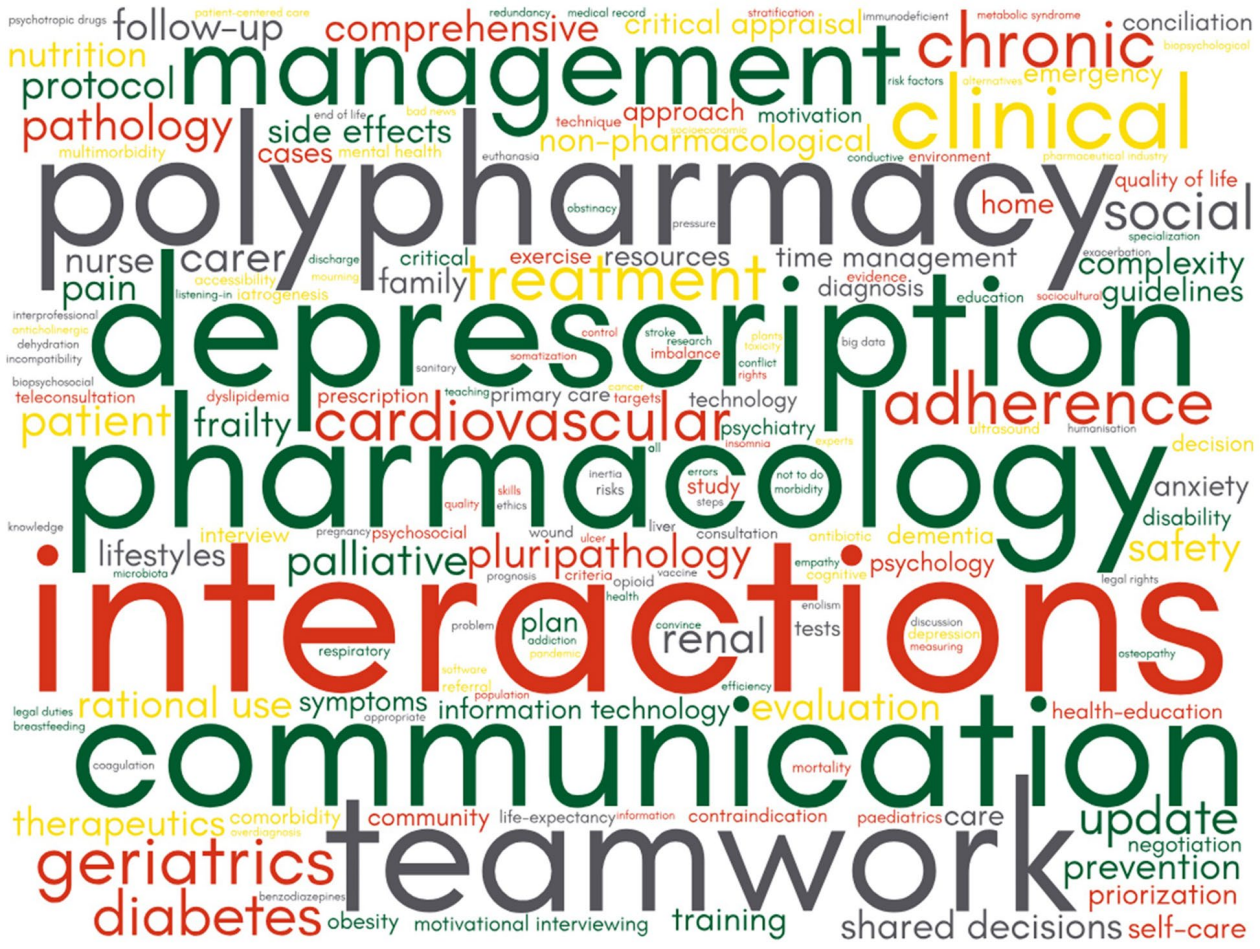
Word cloud of primary care professionals’ training needs

The most frequent terms from responses to the question on training needs are shown in Fig. 5, where word size reflects frequency. The main terms and their counts are also listed in Table 2.

**Table 2:** Most frequent terms identified in open-text responses related to training needs in multimorbidity care (ranked by frequency)

Count (n)	Term
109	interactions
98	pharmacology
91	deprescription
74	communication
62	polypharmacy
51	teamwork
40	clinical
34	chronic
30	geriatrics
29	adherence
29	management
29	treatment
27	cardiovascular
25	pluripathology
19	diabetes
19	palliative

Frequencies indicate the number of occurrences of each term in responses to the open-ended question on perceived training needs

Participants presented their perspectives in more detail for each of the sections (Supplementary Table AF13). Among the difficulties identified, they highlighted lack of time [“*There is not enough time to adequately care for these patients, to engage in shared decision-making, and to work on basic aspects with them*”—physician, 32 years old—] and diagnostic and pharmacological complexity [“*Aligning recommendations for different pathologies with the patient’s needs and the polypharmacy resulting from consultations with different specialists*”—physician, 52 years old—]; and a lack of interprofessional coordination and communication [“*The lack of coordination among all participants in patient care*”—nurse, 57 years old—]. In terms of tools, participants highlighted their commitment to keeping up to date with the scientific literature and to promoting patient well-being by providing the best possible care despite limited resources. [“*I try to update my training and dedicate time to them, even though I am always the last to leave the centre*” —physician, 44 years old—]. Participants also identified experience, clinical guidelines, and interaction with other colleagues [“*Experience and training, as well as coordination with the hospital’s liaison nurses and the municipality’s social services*” —nurse, 53 years old—]. They reported applying organisational skills to adapt care to available resources and maintain quality care [“*Insistence and active patient recruitment, whenever they come to the health centre, we try to do everything possible*” —nurse, 25 years old—].

Regarding learning formats, half of the respondents (50.4%) expressed interest in online courses, followed by blended learning (46.2%) and in-person courses (29.1%). The preferred training materials were texts (88.3%) and videos (84.7%), followed by audio content (32.5%).

**Fig. 6:**
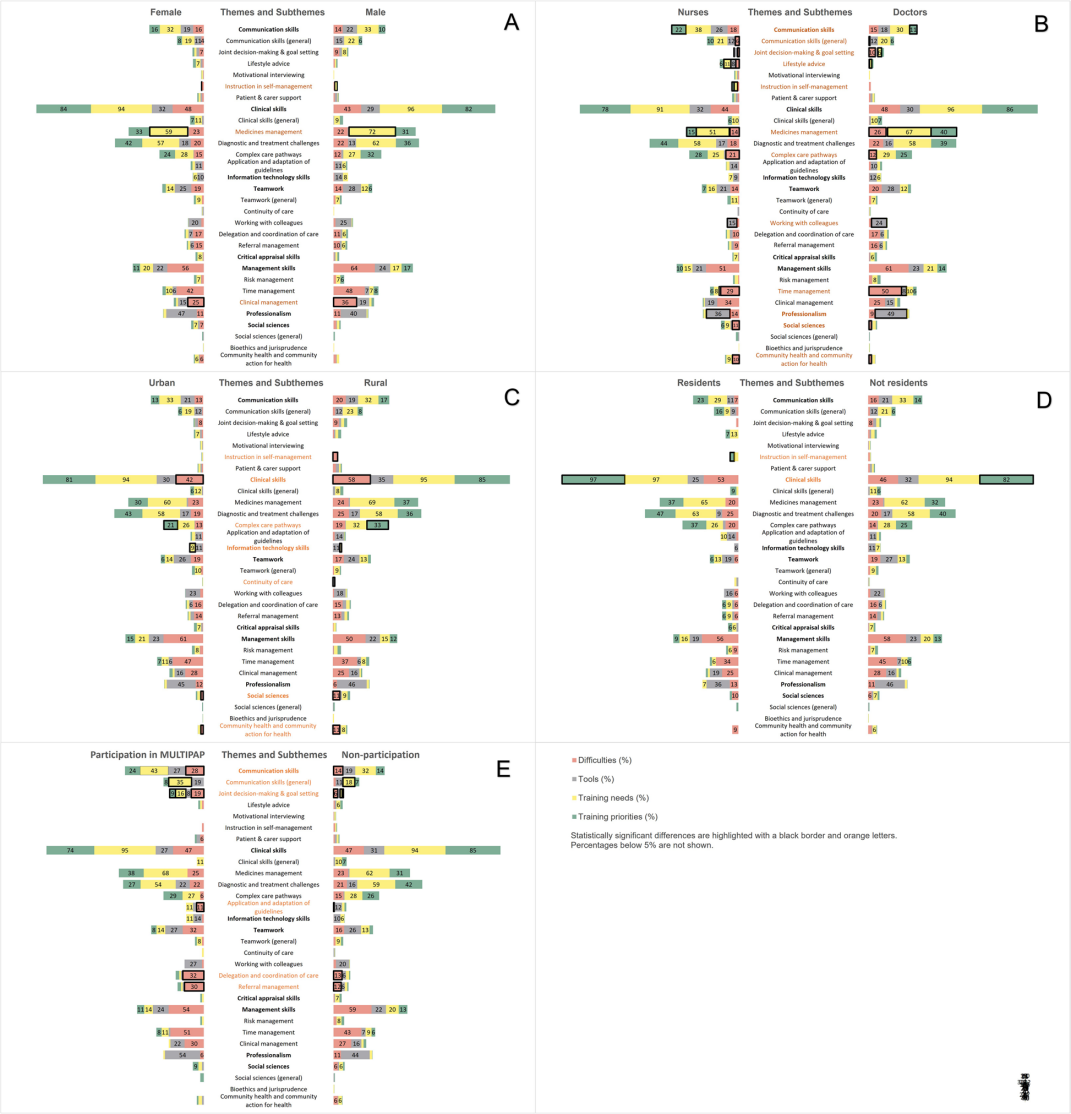
Subgroup analysis of difficulties, tools, needs and training priorities in caring for patients with multimorbidity (**A-E**)

Bivariate analyses showed differences across categories according to profession, gender, work setting, trainee status, and participation in MULTIPAP (Fig. 6 and Supplementary Tables AF14-17). Thus, physicians more frequently reported difficulties related to time management and medicines management, whereas nurses more often referred to complex care pathways and community-related aspects. Participants involved in the prior MULTIPAP training reported communication and coordination difficulties more often than non-participants. Residents prioritized clinical competencies as training needs more frequently, and professionals working in urban settings mentioned, in this sense, information-technology skills more often than those in rural settings. Multivariable analyses were performed to explore these associations after adjustment for potential confounders (see Supplementary Table AF18). Some associations remained statistically significant. Difficulties in time management were less frequently reported by nurses than physicians (aOR 0.42; 95% CI 0.25–0.71), and identification of information-technology skills as a training need was associated with working in an urban setting (aOR 3.52; 95% CI 1.02–12.17). Medicines management training needs remained associated with profession and work setting, and health-promotion needs were more frequently reported by nurses and residents. Participation in prior MULTIPAP study also remained associated with reporting difficulties in communication and coordination.

### MULTIPAP curriculum content proposal

**Fig. 7:**
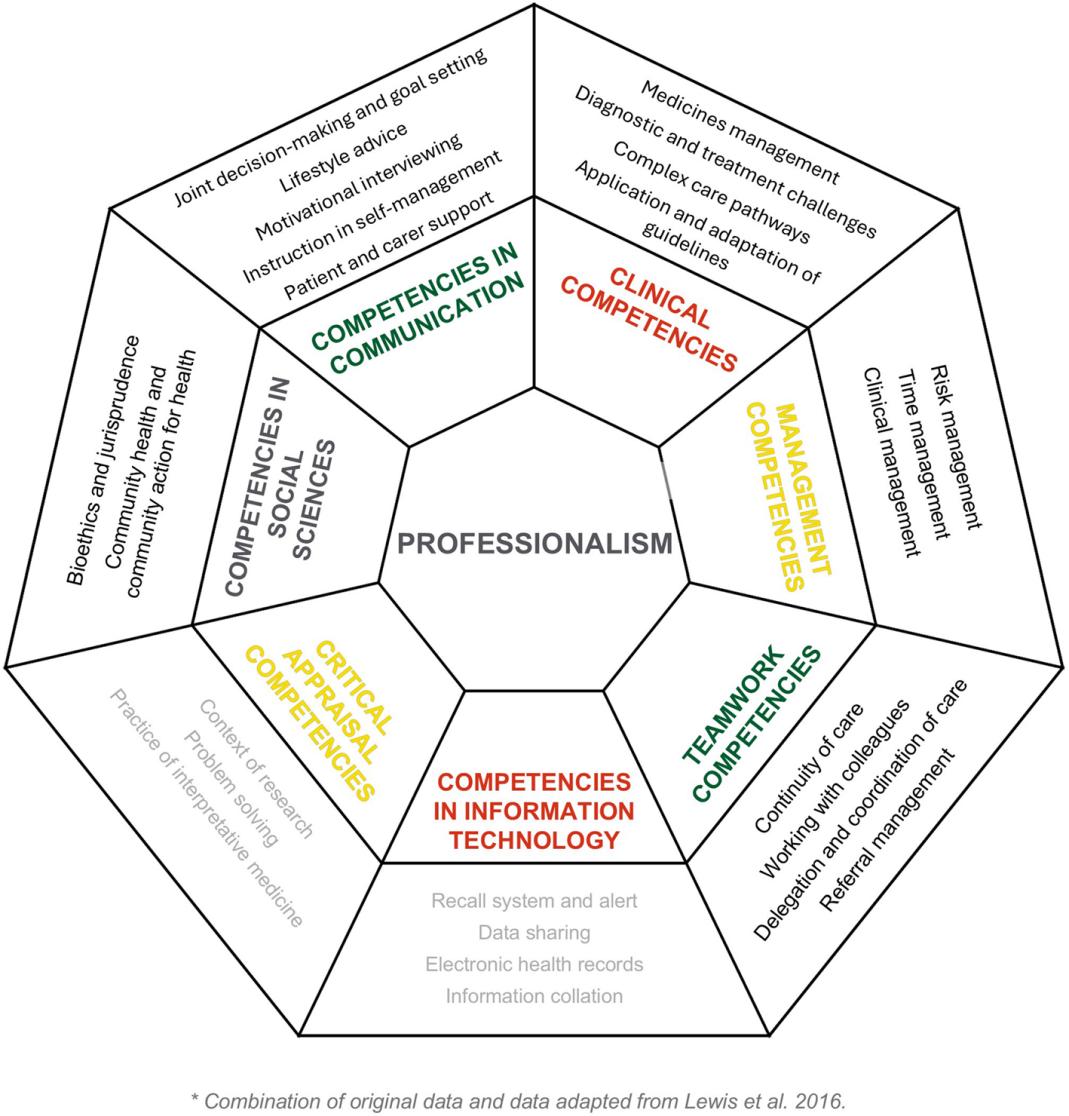
MULTIPAP proposal for multimorbidity curriculum content, based on predefined competencies

We developed updated curricular content (Fig. 7) based on a previous proposal by Lewis et al. [25] incorporating the Ariadne Principles [18], and integrating the new categories identified in this study using an inductive-deductive process based on the codes identified in the analysis of respondents’ open-ended survey answers, which were subsequently grouped according to the categories of the previously established framework (see Methods, Fig. 1). Although no subcategories appeared under "Critical Appraisal" or "Information Technology," these domains were retained to ensure comprehensive training.

## Discussion

The training needs and priorities identified in this survey align with the difficulties encountered by respondents in caring for patients with multimorbidity. Key needs and priorities identified include medicines management and diagnostic and therapeutic issues, followed by communication skills and time management. Professionalism emerged as the main resource used by participants to address the challenges posed by these patients.

Respondents’ training needs, perceived difficulties, and use of tools may vary by profession, field of work, gender, and prior training experience.

These findings are discussed below in relation to the existing literature, with particular attention to the organisational challenges that emerged from our study, the role of professionalism, and the training needs identified by healthcare professionals. An updated curriculum for professionals managing multimorbidity should incorporate previously identified content [25], together with components of professionalism and the social sciences, including community engagement, bioethics, and the legal framework.

### Comparison with existing literature

#### Organisational challenges in managing multimorbidity

The main difficulty identified by participants was a lack of time, in line with the findings of several previous studies [42–48]. Physicians experience more difficulties in time management than nurses, which may be due to a greater administrative burden and more complex clinical decision-making [48–50]. This pressure can translate into stress and dissatisfaction for healthcare professionals and may negatively affect the quality of care [51–53]. The literature proposes several resources to mitigate this barrier, including the prioritisation of patient problems and a progressive approach following an initial assessment of complexity [43, 44, 46]. A holistic and longitudinal perspective of the patient and their context, together with prioritisation and person‑centred care are recognised as key skills of PC professionals [44–46, 54–56] that enable a more efficient model [57]. In short, the essential features of PC [16, 46, 47] appear to support healthcare professionals when managing multimorbidity.

Respondents also highlighted difficulties in coordination and communication across professionals and levels of healthcare, contributing to fragmentation of care. This underscores the need to reconfigure health services in response to multimorbidity [1, 54, 58], incorporating clinical, community, and social dimensions to achieve a holistic and personalized approach [3, 56, 59].

#### The role of professionalism

Professionalism was identified as a key resource for managing multimorbidity, and appeared to be more frequently reported by physicians, although this difference diminished after adjustment, consistent with previous studies showing similar levels among physicians and nurses [60]. In identifying resources to manage multimorbidity, survey respondents highlighted training, experience, empathy, teamwork, and patience, classic traits of healthcare professionalism [61, 62]. Respondents also mentioned the provision of safe care based on the best available evidence, effective communication with team members, patients, and their families, organisational improvements, and the efficient use of resources [17]. Clinical experience was deemed essential and is enhanced by teamwork and interaction with colleagues, which promote shared learning, feedback, and enrichment of the decision-making process [63, 64].

The literature underscores the importance of teaching professionalism to meet healthcare challenges and reinforce professional identity [65, 66]. However, professionalism could conflict with well-being if expectations of excellence are prioritized over healthcare professionals’ health, especially in high-workload settings, calling for organisational measures to prevent burnout [67, 68].

#### Training needs identified by professionals

Medicines management and the handling of clinical complexity were identified as the main training needs, aligning with previous reports [42–44, 55, 56, 69]. Physicians particularly emphasised medicines management, likely because of their prescribing responsibilities. In contrast, nurses prioritised health promotion, reflecting a more salutogenic orientation. These differences align with previous studies showing distinct training demands according to professional profile [70, 71].

Participants also noted the need to strengthen communication skills, reflecting their importance for effective, person-centred care, particularly in the context of multimorbidity [59]. However, implementation of these skills is also restricted by the limited duration of consultations [43, 48, 72].

#### Digital competencies and prior training experiences

A striking finding of our survey was the scarcity of respondents who identified information technology skills as training needs. Resistance to digital tool adoption is well documented [73–75] and contrasts with recommendations emphasizing their role in coordinated, safe, and efficient care; process optimisation; improved decision‑making; treatment personalisation; support for research; patient empowerment; and the reduction of professional inequalities [73, 74, 76]. This need was more frequently identified by professionals in urban versus rural settings, which may reflect the gap in resource availability, access to continuing education, digital infrastructure, and the prioritization of these skills in more remote areas [77].

Finally, participation in prior training initiatives such as the MULTIPAP study [36], which promote critical reflection on clinical practice, increase awareness of the challenges of multimorbidity, and provide tools to identify areas for improvement, appeared to heighten professionals’ awareness of their training needs compared with those without access to this type of training [78, 79].

### Strengths and limitations

This study has several strengths. First is the wide distribution of the online survey (multi-professional, including trainees, nationwide scope). Second, free-text responses to the questionnaire allowed participants to identify training needs, which can be understood as discrepancies, preferences, or shortcomings [80]. Third, the study incorporated strategies to preserve credibility, transferability, dependability, and confirmability [81].

Several limitations should also be noted. First, we used non‑probability convenience sampling, and the recruitment strategy relied on open calls through professional mailing lists and social media, as a result, participation likely favoured digitally engaged and highly motivated professionals. Physicians were somewhat overrepresented and nurses underrepresented, and women slightly overrepresented, reflecting the convenience sampling strategy. Although the geographical coverage of the survey was broad, the distribution of respondents was uneven, with some provinces showing no participation. Consequently, the sample should not be considered statistically representative of all Spanish PC professionals [82], and the findings are best interpreted as exploratory signals to inform educational prioritisation. Second, the influence of the COVID-19 pandemic on our findings remains uncertain. The timing and context of data collection (during a period of high workload and post-pandemic strain in Spanish PC) could have influenced participants’ responses [83]. Service reorganisation and the rapid shift to remote consulting [84, 85] likely increased the perceived importance of skills for safe medicines management and decision-making under uncertainty. Concurrent workload pressures also heightened perceived needs related to time management and communication, while professionalism emerged as a key resource for managing challenging conditions. These perceptions may evolve as organisational circumstances change and relevant training is implemented. In addition, the transferability of the results to other healthcare systems is unknown; while the findings may not be statistically generalisable, they are likely transferable to similar PC contexts with comparable organisational characteristics. Third, the qualitative coding process itself led to some dilution of individual responses through thematic aggregation [86]. Fourth, self-administered surveys may yield less precise results, so combining them with other methods is advisable [87]. Fifth, subgroup differences were initially examined using bivariate analyses and further assessed with multivariable binomial logistic regression to account for potential confounders, although the study was not specifically powered to detect differences between subgroups, and the relatively small number of participants in some groups (particularly residents) may have limited the statistical power and robustness of these comparisons. Finally, the view rate could not be reliably calculated because the number of unique site visitors could not be determined; however, the achieved sample size was adequate for both the quantitative and qualitative analyses conducted.

### Implications for research and practice

This study is among the first in Spain to explore perceived training needs related to the care of patients with multimorbidity among PC physicians and nurses. The study’s design allowed comparison with, and updating of, previous curricular proposals [18, 25], integrating elements of professionalism and the social sciences. While many needs and resources were shared across professionals, some differences may help guide practical adaptations to training programs. Thus, the reported results could act as a context-sensitive guidance that can inform existing training programs, such as the recently approved Family and Community Medicine specialty program [23] in Spain, highlighting competencies that could be prioritized. It may also guide the development and reinforcement of continuing professional education programs through the national health system and professional societies.

Furthermore, it could suggest the development of new, complex interventions to improve outcomes in multimorbidity, which would include, among their components, training contents based on the needs identified and incorporated into the updated curriculum, as well as practical ways to evaluate and refine prior existing interventions through the lens of the updated proposal.

Further studies with larger and more diverse samples, as well as complementary methods such as structured consensus among professionals, are needed to help support these findings and explore, more in depth, their relevance in clinical practice.

## Conclusion

PC physicians and nurses in Spain share several perceived training needs for managing multimorbidity, particularly in medicines management, diagnostic and therapeutic decision-making, communication, and time management. Professionalism emerged as a central resource for addressing the challenges posed by these patients. Variations in training needs and perceived difficulties were observed across professional roles, practice settings, age, gender, and prior experience. These findings offer insights that may help inform the updating of curricula aimed at multimorbidity management in PC. Differences across professional and demographic groups suggest that more tailored educational approaches could be considered and further investigated.

## Supplementary Information

Supplementary Material 1.

## Data Availability

The datasets generated and analysed during the current study are not publicly available due to ethical and legal restrictions related to data protection under the EU General Data Protection Regulation (GDPR) and the conditions of participants’ informed consent, but coded data may be made available from the corresponding author on reasonable request.

## Abbreviations

PC: Primary care
aOR: Adjusted odds ratio
CI: Confidence interval
