# Supplementary material for: Training needs and curricular priorities for managing multimorbidity in Spanish primary care: findings from a national exploratory online survey
Source: BMC Prim Care. 2026 May 6;27:247. doi: 10.1186/s12875-026-03313-6 (PMC13321664; doi:10.1186/s12875-026-03313-6)
Supplement: Supplementary file 1 — Supplementary Material 1. [file 12875_2026_3313_MOESM1_ESM.pdf]

ADDITIONAL FILES (AF1-AF18)

Additional file 1

TABLE AF1. Checklist for reporting results of internet e-surveys (CHERRIES)

Item Category	Checklist Item	Explanation
**Design**	Describe survey design	The survey targeted doctors and nurses working in Primary Care and Out-of-hospital Emergency services within the National Health System of Spain. Participants were recruited using convenience sampling. Convenience sampling may limit the generalizability of the findings.
**IRB (Institutional Review Board) approval and informed consent process**	IRB approval	Approved by Clinical Research Ethics Committee of Malaga
	Informed consent	Participants provided informed consent prior to beginning the questionnaire. Detailed information was provided in the participant information sheet.
	Data protection	No personal data were collected. Anonymous survey setup.
**Development and pre-testing**	Development and testing	An ad hoc questionnaire was developed based on a literature review and hosted in LimeSurvey®. It was piloted among professionals with a profile similar to the target population to ensure clarity and relevance.
**Recruitment process and description of the sample having access to the questionnaire**	Open survey versus closed survey	Open survey
	Contact mode	Introductory message included in the body of emails or posts.
	Advertising the survey	The survey was advertised via emails, mailing lists (MEDFAM), the Community Nursing Association website, personal WhatsApp messages, and generic posts on Facebook and Twitter. A close language style was used in all communications.
**Survey administration**	Web/E-mail	On website (LimeSurvey®)
	Context	Variability in administration sites.<br>A high proportion of invitations were sent to multi-professional teaching units in Community and Family Care, which may include professionals still in training. These participants are presumed to be younger, highly curious, and less experienced, among other factors.
	Mandatory/voluntary	Voluntary
	Incentives	Without incentives
	Time/Date	From November 15, 2021, to July 15, 2022
	Randomization of items or questionnaires	Yes
	Adaptive questioning	Yes
	Number of Items	The largest number of items was 20 on page 2 of 4
	Number of screens (pages)	4
	Completeness check	All items included a non-response option ("others"), and selection of one response option was enforced. Completeness was verified using JavaScript before submission. Null responses to open-ended questions were identified and removed during the survey analysis phase.
	Review step	Participants could review and edit answers using a "Previous" button. Incomplete surveys could be saved and resumed later; however, responses could not be modified after submission.
**Response rates**	Unique site visitor	Based on cookies set in the participant's browser.
	View rate (Ratio of unique survey visitors/unique site visitors)	Could not be calculated because the number of unique site visitors cannot be reliably determined.
	Participation rate (Ratio of unique visitors who agreed to participate/unique first survey page visitors)	850/2615 = 32.5%
	Completion rate (Ratio of users who finished the survey/users who agreed to participate)	387/850 = 45.5%
**Preventing multiple entries from the same individual**	Cookies used	Yes, cookies are enabled to prevent multiple responses from the same browser.
	IP check	Not applicable
	Log file analysis	Not applicable
	Registration	Not applicable
**Analysis**	Handling of incomplete questionnaires	Only completed questionnaires were analysed, except that qualitative responses from incomplete questionnaires were included in the qualitative analysis.
	Questionnaires submitted with an atypical timestamp	Questionnaires were analysed regardless of their submission timestamp.
	Statistical correction	No statistical corrections were applied to account for potential non-representativeness of the sample.

This table has been modified from Eysenbach G. Improving the quality of Web surveys: the Checklist for Reporting Results of Internet E-Surveys (CHERRIES). J Med Internet Res [Internet]. 2004 [cited October 30, 2025];6(3):e34. Available in: https://www.jmir.org/2004/3/e34/; erratum available https://www.jmir.org/2012/1/e8/. Copyright ©Gunther Eysenbach. Originally published in the Journal of Medical Internet Research, 29.9.2004 and 04.01.2012.

Additional file 2a:

AF2a - Study-specific questionnaire (English version)

TRAINING NEEDS ASSESSMENT OF PRIMARY CARE PROFESSIONALS IN MULTIMORBIDITY

This anonymous survey aims to identify and analyze the main tools and limitations of primary care physicians and nurses in the management of patients with multimorbidity, in order to design training interventions to improve clinical practice.

Page 1: INFORMED CONSENT

View the participant information sheet

Welcome to the questionnaire of the project “Identification of training needs of primary care professionals in multimorbidity”.

This questionnaire is addressed to physicians and nurses who routinely work in health centres and/or out-of-hospital emergency services of the Spanish National Health System.

I have read the participant information sheet (link provided at the beginning of this section) and understand the formal aspects involved. My participation consists of completing an ANONYMOUS questionnaire after providing informed consent. Participation is VOLUNTARY, and I may request the withdrawal of my data at any time. I will NOT receive any financial compensation. The information collected will be treated confidentially. I know how to contact the research team.

Please select the option below to indicate your consent to participate in the study
[ ] I voluntarily agree to participate in the study "Identification of training needs of primary care professionals in multimorbidity"

Page 2: QUESTIONS

Questions marked with an asterisk (*) are mandatory

1. **Channel through which you received this survey ***
   Please select only one of the following options:
   - o Institutional email
   - o Non-institutional email
   - o Social networks
   - o Instant messaging

2. **Province where you currently work***
   Please write your answer here:

3. **Current profession***
   Please select only one of the following options:
   - o Physician
   - o Nurse
   - o Family Medicine resident
   - o Community Nursing resident
   - o Other (please specify):

4. **Current professional role***
   Please select only one of the following options:
   - o Primary Care team
   - o Primary Care emergency services
   - o Both
   - o Other (please specify):

5. **Age***
   Please write your answer here:

6. **Gender***
   Please select only one of the following options:
   - o Male
   - o Female
   - o Prefer not to answer
   - o Other (please specify):

7. **Main work setting***
   Please select only one of the following options:
   - o Urban
   - o Rural
   - o Both

8. **Have you received any training in multimorbidity?***
   Please select only one of the following options:
- o Yes
- o No

9. **Type of training received**
   Please select only one of the following options:
- o Face-to-face course
- o Online course
- o Single talk or seminar
- o Self-training
- o Other (please specify):

10. **When did you complete this training?**
    Please select only one of the following options:
- o Within the last year
- o More than 1 year but less than 5 years ago
- o More than 5 years ago
- o Other (please specify):

11. **Duration of training**
    Please select only one of the following options:
- o 30 hours or more
- o Less than 30 hours

12. **Training content**
    Please check all that apply:
- o Communication skills
- o Clinical skills
- o Management skills
- o Critical appraisal skills
- o Teamwork
- o Information technology skills
- o Other (please specify):

13. **Have you participated in the MULTIPAP Study?***
    Please select only one of the following options:
- o Yes
- o No

14. **Please write three perceived training needs related to the care of patients with multimorbidity and polypharmacy.**
    Please write your answers here:
    1.
    2.
    3.

15. **If you were offered a course on multimorbidity next month, what would you like it to focus on?***
    Please write your answer here:

16. Preferred learning format*

Please check all that apply:

- Face-to-face courses
- Blended courses
- Online courses
- Other (please specify):

17. Preferred learning materials*

Please check all that apply:

- Videos
- Texts
- Audio
- Other (please specify):

18. What are the main difficulties and challenges you currently face in caring for patients with multimorbidity?*

Please write your answer here:

19. What are your main tools and resources to address these difficulties?*

Please write your answer here:

20. Which definition of multimorbidity do you consider most accurate?*

Please select only one of the following options:

- The presence of a chronic disease in combination with at least one additional disease (acute or chronic), biopsychosocial factor, or somatic risk factor within the same individual (European General Practice Research Network)
- The coexistence of two or more chronic health conditions in the same individual (WHO)
- Coexistence of three or more chronic conditions (Fortin et al., 2012)
- Other (please specify):

Page 3: SKILLS ASSESMENT

Please indicate the importance you attribute to the following skills in daily clinical practice, from 1 (no importance) to 4 (maximum importance).

Please select the appropriate answer for each item:

	1	2	3	4
Communication – support for patients and/or caregivers	O	O	O	O
Pharmacological treatment management	O	O	O	O
Risk management	O	O	O	O
Electronic record keeping and data collection	O	O	O	O
Critical appraisal for research and problem solving	O	O	O	O
Implementation and adaptation of clinical practice guidelines	O	O	O	O
Communication – promotion of healthy lifestyles and self-care	O	O	O	O
Patient-centred care	O	O	O	O
Shared decision-making	O	O	O	O
Time management	O	O	O	O
Teamwork – continuity and coordination of care	O	O	O	O
Care plan design	O	O	O	O

Page 4: FINAL COMMENTS

Please leave any comments or suggestions regarding the questionnaire or the project. Thank you very much for your collaboration.

The questionnaire has been completed. **Don’t forget to click “Submit” in the bottom-right corner.**

Thank you very much for your participation—your responses are greatly appreciated!

It would also be very helpful if you could share this survey with colleagues who might be able to respond.

If you have any comments or suggestions regarding the questionnaire or the project, please let us know by writing them in the box below.

Additional file 2b:

TABLE AF2b. Summary of survey variables, types and definitions

Variable	Type / Format	Definition / Categories
**Survey administration**		
Survey distribution channel	Categorical	Channel through which the questionnaire was sent or received: Institutional email / Non-institutional email / Social networks / Instant messaging
**Sociodemographic characteristics**		
Province of origin	Open-ended	Free-text
Age	Numeric	Respondent's age at the time of the survey
Gender	Categorical	Male / Female / Prefer not to answer / Other (specify)
**Professional characteristics**		
Profession	Categorical	Doctor / Nurse / Family Medicine Resident / Community Nursing Resident / Other (specify)
Current professional role	Categorical	Primary Care team / Primary Care Emergency Service / Both / Other (specify)
Work setting	Categorical	Rural / Urban / Both
**Training in MM**		
Specific training in MM	Dichotomous	Yes / No
Type of MM training received	Multiple choice	Face-to-face course / Online course / Single talk or seminar / Self-training / Other (specify)
Timing of MM training	Ordinal	Last year / 1–5 years ago / More than 5 years ago / Other (specify)
Length of MM training	Ordinal	Less than 30 hours / 30 hours or more
MM training content	Multiple choice	Communication skills / Clinical skills / Management skills / Critical appraisal skills / Teamwork / Information Technology skills / Other (specify)
**Participation in projects**		
Participation in MULTIPAP Project	Dichotomous	Yes / No
**Perceptions and needs**		
Perceived training needs	Open-ended	Free-text
Preferred topics for future multimorbidity training	Open-ended	Free-text
Desired learning format	Multiple choice	Face-to-face / Blended / Online / Other (specify)
Desired learning materials	Multiple choice	Videos / Texts / Audios / Other (specify)
**Clinical practice challenges and resources**		
Main challenges in care of patients with MM	Open-ended	Free-text
Tools and resources used	Open-ended	Free-text
**Conceptual definitions**		
Definition of MM	Categorical	The presence of a chronic disease in combination with at least one additional disease (acute or chronic), biopsychosocial factor, or somatic risk factor within the same individual / The coexistence of two or more chronic health conditions in the same individual / Coexistence of three or more chronic conditions / Other (specify)
Perceived importance of skills (12 items)	Likert-type (ordinal)	Adapted from Lewis et al., 2016: shared decision-making, time management, designing care plans, teamwork, supporting patients/caregivers, pharmacological management, risk management, information technology use, critical appraisal, guideline application, healthy lifestyles, patient-centred care

MM: multimorbidity

Additional file 3: TABLE AF3. Code correspondence across researchers 1 and 2 (AGH and FLF). Detailed codes for researcher 1 (FLF) are available in Table AF4.

	AGH	FLF										
**Tools**												
Shared information/medical record	C1	C138	C58									
Information search/study/self-training/update/case studies	C2	C181										
Internet/online resources	C3	C181	C58									
Consultation management/margin for management/time management	C4	C20	C182	C190	C193	C188	C174					
Work protocols/clinical guidelines	C5	C19	C17	C21								
Study/self-training/update/case study	C6	C181	C185									
Team spirit/teamwork	C7	C9	C82	C189	C45							
Communication skills	C8	C27	C2	C91	C108	C7	C105					
Teamwork/multidisciplinary healthcare/coordination/primary care team	C9	C9	C45	C82	C104							
Prescription/deprescription tools	C10	C101	C8	C21	C58	C18	C46					
Specialized health training/continuing training	C11	C192	C114	C186	C181							
Common sense	C12	C185										
Longitudinality	C13	C183	C82	C83	C160							
Patience	C14	C185	C2									
Patient trust/professional-patient bond	C15	C185	C83	C82	C183	C184						
Information technologies/technologization	C16	C58	C181	C174								
Reference professional	C17	C182										
Desire/extra/outside working hours/goodwill/motivation/professionalism	C18	C185	C189	C181								
Ultrasound	C19	C97	C10									
Clinics	C20	C10	C51	C28	C6	C51						
Evidence-based medicine and independent bibliography	C21	C59	C181	C101								
Home visit	C22	C144	C44									
Scheduled consultations/chronic consultations	C23	C6	C83	C99	C113	C182						
Knowledge of the context/biopsychosocial approach	C24	C47	C111	C115	C149	C37	C49	C134	C183	C156	C74	C32

(continuation)

	AGH	FLF						
Integrality/comprehensive approach	C25	C82	C74	C36	C71			
Experience/trial and error	C26	C187						
Clinical referrals	C27	C135	C45	C49				
Patient follow-up	C28	C83	C160					
Methodology/systematization/planning	C29	C19	C21	C17	C35	C182	C109	C127
Health education	C30	C25	C3	C52	C24			
Decision reversibility	C31	C83	C183	C31				
Development of care plans	C32	C19	C17	C113	C20	C64		
Imagination/creativity	C33	C185						
Conferences	C34	C181	C186	C114				
Questionnaires/tests/assessment/index and health measurement scales	C35	C21	C61	C50				
Empathy	C36	C185	C2					
Accessibility	C37	C78	C82	C134				
Training (general)	C38	C192	C114	C186	C181	C51		
Patient-centered care	C39	C111	C127	C156	C31			
Collaboration with hospital	C40	C45	C135					
Anticipation	C41	C185	C187	C31				
Tutoring	C42	C191						
Presence/contact	C43	C174						
Prudence/minimally disruptive practice/quaternary prevention	C44	C102	C23	C112	C46	C72		
Rurality	C45	C194						
Drugs and diagnostic tests	C46	C46	C18	C4	C5	C96	C40	C21
Sufficient resources/material	C47	C79	C139					
Group dynamics	C48	C32	C108	C24				
Communication, synergy and relationship with family and caregivers	C49	C184	C62	C7	C108			
Not codable	NC	NC						

(continuation)

	AGH	FLF									
**Difficulties**											
Time	C1	C20	C163	C190							
Phone	C2	C174									
Professional demotivation	C3	C178	C43	C176							
Human resource shortage	C4	C175	C170	C79	C139						
High demand	C5	C99	C177	C20	C169	C172	C168				
Poor coordination	C6	C45	C9	C168							
Interactions and side effects	C7	C128	C145	C4	C70	C38	C12	C23			
Lack of training	C8	C114	C165	C186	C192	C51					
Polypharmacy and pharmacology	C9	C1	C23	C18	C38	C96	C5	C46			
Complications	C10	C92	C28	C83							
Pandemic	C11	C67									
Lack of longitudinality and difficulties in follow-up	C12	C169	C82	C83	C176						
Lack of comprehensive, biopsychosocial and holistic approach	C13	C115	C47	C37	C79	C74	C168				
Patient and family passivity	C14	C184	C22	C62	C64	C108					
Complexity	C15	C95	C171	C28	C98	C84	C6	C80	C11	C182	C63
Communication related problems	C16	C20	C27	C91	C184	C108	C2	C7	C150	C31	C169
Lack of specialized training	C17	C192	C165	C114	C186						
Scarce resources	C18	C170	C79	C139	C175	C176	C163	C164			
Lack of adapted clinical guidelines	C19	C17	C40	C35	C171	C180	C23	C109	C83	C84	C28
Delay in receiving care	C20	C172	C168	C78							
Therapeutic inertia	C21	C136									
Ageing	C22	C167	C41	C30	C34	C110					
Social determinants of health approach	C23	C149									
Care and self-care deficit	C24	C22	C64	C104	C62						

(continuation)

	AGH	FLF				
Information overload	C25	C138				
Decision making	C26	C31	C127			
Bureaucracy	C27	C168				
Home visit	C28	C144	C44			
Computing and new technologies	C29	C170	C138	C58		
Family conciliation	C30	C179	C176	C168		
Bad management	C31	C20	C168			
Barriers to accessibility	C32	C169	C78	C177	C173	
Hospitalocentrism	C33	C45				
Pharmaceutical industry	C34	C101				
Lack of critical evaluation	C35	C59				
Lack of recognition	C36	C166	C43	C178	C168	
Defencelessness	C37	C166				
Little privacy	C38	C150	C164			
Deprescription	C39	C8	C96	C72	C1	C46
Low involvement	C40	C165				
Technologization	C41	C58	C138	C170		
Disorganization	C42	C176	C168			
Pressure on healthcare	C43	C172				
No adhesion	C44	C26				
Lack of social health resources	C45	C149	C37	C47	C49	C170
Fragility	C46	C167	C41	C30	C34	C6
Poverty	C47	C149				
Inequities	C48	C149	C102			
Don't do	C49	C112	C23			

(continuation)

	AGH	FLF											
Changing habits	C50	C29	C108	C7									
Iatrogeny	C51	C102	C112	C23	C72								
Patient and family expectations	C52	C184	C161	C127	C62	C31							
Health education	C53	C52	C25	C3									
Mental health	C54	C66	C105	C47	C118								
Quality of life	C55	C73											
Applicability	C56	C169											
Not codable	NC	NC											
**Training needs and training priorities**													
Polypharmacy	C1	C1	C85	C88									
Deprescription	C2	C8	C23	C96									
Interactions	C3	C4	C7	C128	C145	C70							
Adherence to treatment	C4	C26											
Side and adverse effects	C5	C38	C85	C16									
Pharmacotherapeutic optimization/contraindications	C6	C23	C8	C96	C46	C5	C128	C109	C38	C123	C156		
Time and time management	C7	C20	C163	C190	C168	C54	C99						
Communication	C8	C2	C91	C184	C108	C27	C7	C150					
Biopsychosocial model and community approach	C9	C32	C47	C115	C37	C139	C49	C149	C150	C79	C74	C105	C134
Teamwork and interprofessional coordination	C10	C9	C49	C45	C151	C129							
Pharmacology	C11	C18	C53	C23	C39	C93	C89	C103	C107	C88	C122	C42	
Health prevention and promotion (including health education)	C12	C3	C25	C30	C52	C104							
Diabetes	C13	C56											
Home care	C14	C144	C44	C119									

(continuation)

	AGH	FLF													
Geriatrics	C15	C41	C36	C30	C34	C141	C167								
Cognitive functions and dementia	C16	C13	C61	C66	C157	C41									
Kidney and liver problems	C17	C12	C68												
Heart disease	C18	C69	C71	C94	C60	C57									
End of life and palliative care	C19	C106	C14	C75	C86	C155	C121	C110	C10						
New technologies and information technologies	C20	C58	C138												
Clinical skills	C21	C10	C98	C95	C80	C40	C63	C92	C117	C152	C87	C51	C133	C147	C148
Clinical guidelines, protocols, standardized actions and scales	C22	C17	C19	C124	C21	C83	C151	C117	C35	C36	C113	C125	C142		
Pain	C23	C15	C93												
Specific aspects in multimorbidity, polypathology and chronic patient	C24	C51	C28	C6	C95	C40	C152	C80	C148	C11	C35	C84			
Caregivers and family members	C25	C62	C184	C161											
Obesity and metabolic syndrome	C26	C116	C131												
Management and planning skills	C27	C20	C168	C54	C99	C83	C158	C188	C84	C79	C113				
Respiratory pathology	C28	C55	C159												
Mental health	C29	C66	C105	C118	C93	C61	C13	C65	C140	C166	C2	C132			
Shared decision making and patient-centered care	C30	C31	C111	C127	C156										
Patient safety	C31	C72													
Integrality	C32	C74	C82	C119	C36										
Research/Evidence-based medicine/training and updating of professionals	C33	C137	C5	C181	C114	C186	C192	C59	C51	C50	C101	C129			
Lifestyle and habits	C34	C29													
Don't do/quaternary prevention	C35	C112	C102	C100	C123	C40									
Quality of life	C36	C73													
Primary care	C37	C82	C119												

(continuation)

	AGH	FLF					
Accessibility	C38	C78	C82	C119			
Ultrasound	C39	C97					
Polypharmacy and pharmacology	C40	C101					
Clinical case	C41	C81					
Pregnancy, lactation and childhood	C42	C90	C126				
Ethical and legal aspects	C43	C154	C76				
Oncology	C44	C130					
Inertia	C45	C136					
Continuity and patient follow-up	C46	C160	C51	C83			
Teaching	C47	C191					
Professional motivation	C48	C108	C178	C43			
Immunosuppression	C49	C125					
COVID pandemic	C50	C67					
Digestive pathology	C51	C162					
Non-pharmacological care and measures	C52	C24	C64	C104	C48	C29	C146
Self-care	C53	C22	C48				
**D**ressings	C54	C77	C104	C153			
Nutrition and diet	C55	C33	C146				
Resources	C56	C79					
Not codable	NC	NC					

Additional file 4:

TABLE AF4. Codes from reviewer 1 (FLF)

Polypharmacy	C1
Communication	C2
Health prevention and promotion	C3
Drug interactions	C4
Indications for drugs with evidence	C5
Multipathological management (serious, safe drugs...)	C6
Motivational interview	C7
Deprescription	C8
Teamwork	C9
Clinical skills	C10
Comorbidities	C11
Kidney failure and treatments	C12
Actions in patients with dementia	C13
Palliative care	C14
Chronic pain treatment	C15
Anticholinergic load	C16
Clinical practice guides	C17
Pharmacotherapy	C18
Protocols	C19
Management (time...)	C20
Measuring tools	C21
Self-care	C22
Treatment adequacy	C23
Non-pharmacological therapies	C24
Nurse education	C25
Therapeutic adherence	C26
Active listening	C27
Actions in patients with multimorbidity	C28
Lifestyles	C29
Prevention in elderly patients	C30
Shared decision making	C31
Community care	C32
Nutritional care	C33
Fragility care	C34
Pathology prioritization	C35
Comprehensive geriatric assessment	C36
Dependency care (Social resources...)	C37
Drug side effects	C38
Antibiotic resistance	C39
Diagnostic particularities	C40
Geriatric syndromes	C41
Medicinal plants	C42

Skills to motivate professionals	C43
Home management of acute problems	C44
Relationship between levels of healthcare	C45
Rational use of medication	C46
Psychosocial help	C47
Other ways to take care of yourself	C48
Socio-health coordination	C49
Assessment	C50
Update on chronic pathologies	C51
Health education	C52
Discontinued treatments	C53
Efficiency	C54
Chronic obstructive pulmonary disease	C55
Diabetes	C56
High blood pressure	C57
Information and communications technologies (for clinical follow-up...)	C58
Critical reading	C59
Arrhythmias	C60
Cognitive assessment	C61
Caregivers (training, care)	C62
Urgent situations management	C63
General care	C64
Addictions	C65
Mental health	C66
Assistance in pandemic	C67
Liver diseases and treatments	C68
Cardiovascular diseases	C69
Interactions with osteopathy treatments	C70
Comprehensive treatment of cardiovascular risk factors	C71
Patient safety	C72
Quality of life	C73
Comprehensive approach	C74
Effort limitation	C75
Legal rights	C76
Bed sores	C77
Accessibility	C78
Health and non-health resources	C79
Attention to the most frequent pathologies	C80
Clinical cases	C81
Primary Care	C82
Pathology follow-up	C83
Attention for problems	C84
Therapeutic waterfall	C85
Vital prognosis	C86

Warning signs	C87
Drug combinations	C88
Therapeutic news	C89
Pregnancy and lactation	C90
Bad news management	C91
Decompensations management	C92
Psychotropic drugs	C93
Heart failure	C94
Comprehensive care for complex patients	C95
Treatment reconciliation	C96
Ultrasound	C97
Clinical care for multiple symptoms	C98
Scheduled and on-demand Consultation	C99
Overdiagnosis	C100
Prescription and relationship of the professional with pharmaceutical industry	C101
Quaternary prevention	C102
Most prevalent treatments	C103
Nursing care	C104
Psychological approach	C105
End of life care	C106
Drugs and dehydration	C107
Patient motivation strategies	C108
Treatment prioritization	C109
Life expectancy	C110
Patient centered care	C111
Recommendations don't do	C112
Work in stages	C113
Training	C114
Social valuation	C115
Obesity	C116
Therapeutic objectives	C117
Psychological therapies	C118
Family medicine	C119
Management in risk situations	C120
Euthanasia	C121
Symptomatic treatment	C122
Overtreatment	C123
Evaluation scales	C124
Immunodepression	C125
Paediatrics	C126
Prioritization of patient needs	C127
Disease-drug interactions	C128
Rotations by coordination units	C129
Actions in patients with cancer	C130

Metabolic syndrome	C131
Enolism	C132
Electrocardiogram for nursing	C133
Elimination of sociocultural barriers	C134
Derivations	C135
Therapeutic inertia	C136
Investigation	C137
Information systems	C138
Local resources	C139
Insomnia management	C140
Agitation management	C141
Stratification (risk...)	C142
Dyslipidemia	C143
Home care	C144
Disease-disease interactions	C145
Diet and treatments	C146
Admission prevention	C147
Most prevalent pathologies	C148
Socioeconomic determinants	C149
Humanization	C150
Transition to discharge	C151
Disease exacerbations	C152
Wounds	C153
Ethics	C154
Mourning	C155
Individualization	C156
Neurological diseases	C157
Economic analysis	C158
Dyspnoea	C159
Healthcare continuity	C160
Family-focused care	C161
Digestive pathology	C162
Lack of time	C163
Lack of space	C164
Lack of professional training	C165
Lack of institutional support for professionals	C166
Population aging	C167
Inadequate healthcare organization	C168
Difficulty applying features of anatomical pathology	C169
Insufficient resources (computer…)	C170
Difficulty in interpreting complementary tests	C171
High healthcare pressure	C172
Architectural barriers	C173
Different modalities of healthcare (in person, telephone...)	C174

Lack of professionals	C175
Labor problems (contracts…)	C176
Less patient attendance	C177
Lack of motivation in professionals	C178
Difficulty in personal conciliation of the professional	C179
Variability in clinical practice	C180
Self-learning (information search...)	C181
Organizational improvements in healthcare	C182
Longitudinality	C183
Improve the relationship with the patient/family	C184
Professional with positive attitude/emotions towards healthcare	C185
Continuing training for professionals	C186
Clinical experience	C187
Active recruitment with patients	C188
Professionalism	C189
Time	C190
Teaching role of the professional	C191
Specialized health training	C192
Low healthcare pressure	C193
Rural environment	C194
Non-codable	NC

Additional file 5:

TABLE AF5. Glossary - Theme and subtheme definitions*

THEMES and subthemes	Definitions
COMMUNICATION COMPETENCIES	The efficient transmission of information, including verbal communication (such as speech and listening strategies) and non-verbal communication (such as gestures, facial expressions, eye contact, and body language). These skills enable patients to understand and process information provided by health professionals through empathy, informed collaborative choices, and patient involvement. When patient-centred, communication skills help health professionals identify needs, plan treatment, and create a therapeutic and supportive environment that promotes shared decision-making, treatment adherence, and positive behaviour change (1).
Joint decision-making and goal setting	- Joint decision making: a collaborative process that involves a person and their healthcare professional working together to reach a joint decision about care. This could be immediate care or care in the future, for example through advance decision planning. It involves choosing tests and treatments based on both evidence and the person's individual preferences, beliefs, and values. It means making sure that the person understands the risks, benefits and possible consequences of different options through discussion and information sharing (2).<br>- Collaborative goal setting: process by which caregiver and patient agree on a health-related goal (3).
Lifestyle advice	Health promotion is the process of enabling people, individually and collectively, to increase control over the determinants of health and thereby improve their health. It not only embraces actions directed at strengthening the skills and capabilities of individuals, but also action directed towards changing social, environmental and economic determinants of health so as to optimise their positive impact on public and personal health (4).
Motivational interviewing	Motivational interviewing is a collaborative conversation style whose purpose is to reinforce the person's motivation and commitment to change (5).
Instruction in self-management	Self-management education in multimorbidity is any form of formal education or training for people with long-term conditions focused on helping them to develop the knowledge, skills and confidence they need to manage their own health care effectively (6).
Patient and carer support	- Support: the name of the action through which protection or assistance is given to something or someone, to help them to achieve some objective or to assist something planned to happen. This support can be of different types, and can be materialized in different ways, from economic to emotional, and others (7).<br>- Patient: an individual who seeks care or receives health care due to illness, injury, to improve his or her well-being, to prevent illness or to obtain diagnoses about his or her health status (8).<br>- Caregiver: a person who gives care to people who need help taking care of themselves (9).<br>Informal caregiver: a person who provides some type of unpaid, ongoing assistance with activities of daily living or instrumental activities of daily living to a person with a chronic illness or disability (10).<br>- Formal care: usually refers to paid care services provided by a healthcare institution or individual for a person in need (11).
CLINICAL SKILLS	- Clinical skills are the tangible, practical abilities that healthcare practitioners utilise to directly care for their patients. These skills encompass a range of hands-on procedures and tasks that are essential to the day-to-day management of patient health. They are the embodiment of a practitioner's capacity to apply theoretical knowledge in a clinical setting, and they form the foundation of any healthcare professional's toolset (12).<br>- The authors consider the Miller's pyramid: at the base, knowledge (knows); followed by competence (knows how); above, performance (shows how) and at the top, action (does) (13).
Medicines management	The clinical, cost-effective, and safe use of medicines to ensure that patients gain the maximum benefit from the medicines they need, while minimizing potential harm across all elements of the medicines pathway, from acquisition and storage to prescribing, supply, and administration (14).

Diagnostic and treatment challenges	- Challenge: a new or difficult task that tests someone's ability and skill (15).<br>- Diagnosis: the process of identifying a disease, condition, or injury from its signs and symptoms. A health history, physical exam, and tests, such as blood tests, imaging tests, and biopsies, may be used to help make a diagnosis (16).<br>- Treatment: set of measures and strategies whose main objective is to cure, alleviate or prevent diseases, conditions or symptoms in a patient (17).
Complex care pathways	- Care pathway: a complex intervention for the mutual decision making and organisation of care processes for a well-defined group of patients during a well-defined period (18).<br>- Complex chronic patient: those who are more difficult to manage as they present changing needs that require continuous re-evaluations and make it necessary to use various levels of care in an orderly manner and, in some cases, health and social services (19).
Application and adaptation of guidelines	- Clinical Practice Guideline: set of recommendations based on a systematic review of the evidence and on the evaluation of the risks and benefits of the different alternatives, with the aim of optimizing healthcare for patients (20).<br>- Guideline implementation: the uptake and incorporation of guideline recommendations into practice by the target end users (21).<br>- Guideline Adaptation: a systematic approach to using and adjusting existing guidelines produced in one setting for use in a new setting with a different cultural or organizational context. The process of adapting a guideline and its recommendations must ensure that the adapted guideline addresses specific health questions relevant to the context of use and that it is suited to the needs, priorities, legislation, policies, and resources in the new target setting (21).
MANAGEMENT SKILLS	A set of competencies essential for healthcare professionals to effectively and efficiently manage medical, nursing, and public health resources in order to achieve goals aligned with improving the overall health of both the population and the healthcare system (22).
Risk management	Risk management in healthcare: the clinical and administrative systems, processes, and reporting mechanisms used to identify, monitor, assess, mitigate, and prevent risks in healthcare settings (23).
Time management	A form of decision making used by individuals to structure, protect, and adapt their time in response to changing conditions conditions (24).
Clinical management	An improvement strategy that systematizes and organizes healthcare processes in an appropriate and efficient manner, supported by the best available scientific evidence and involving health professionals in management and decision-making regarding patient care (25).
TEAMWORK	The combined actions of a group of people working together effectively to achieve a goal.<br>An effective team is one where the team members, including the patient, communicate with one another, as well as combining their observations, expertise and decision-making responsibilities to optimize care (26).
Continuity of care	Continuity of care can be defined as the extent to which a person experiences an ongoing relationship with a clinical team, or member of a clinical team. It means coordinated clinical care, that progresses smoothly as the patient moves between different parts of the health service. It can consist of relational continuity – seeing the same people or team, management continuity – management and coordination of care and informational continuity – continuity of patient records and information (27).
Working with colleagues	Work colleagues: people with whom you carry out work tasks or share your work environment. More broadly, colleagues are individuals who share your profession but may work in other organizations, whereas coworkers are those who work with you within the same company or institution (28).
Delegation and coordination of care	- Delegation of care: the transfer of responsibility for performing a specific task from one individual to another, while retaining overall accountability for the outcome (29).<br>- Care coordination: The deliberate organization of patient care activities among two or more participants (including the patient) involved in a patient’s care to facilitate the appropriate delivery of health care services. Organizing care involves aligning personnel and other resources needed to carry out all required patient care activities and is often achieved through the exchange of information among participants responsible for different aspects of care (30).

Referral management	Referral management is a way of monitoring, directing and controlling patient referrals, with the aim of ensuring that the most clinically effective and cost-effective outcomes are achieved, whilst at the same time respecting patients' rights to choice (31).
CRITICAL APPRAISAL COMPETENCIES	Critical Appraisal is the process of carefully and systematically examining research to judge its trustworthiness, and its value and relevance in a particular context. It is an essential skill for evidence-based medicine because it allows people to find and use research evidence reliably and efficiently (32).
INFORMATION TECHNOLOGY COMPETENCIES	- Competency refers to the demonstrated ability to apply knowledge, skills, and personal, social, and/or methodological abilities in work or study contexts, as well as in professional and personal development (33).<br>- Information and Communications Technology: Any equipment or interconnected system used for the automatic acquisition, storage, processing, movement, control, display, exchange, transmission, routing, or reception of data or information. This includes computers, peripheral equipment, system software, support services, and related resources (34).
PROFESSIONALISM	- Professionalism is a multidimensional concept manifested through the knowledge, attitudes, and behaviours that underpin successful clinical practice (35).<br>- It is understood as a dynamic construct, developed within specific interactions and evolving according to the context, clinical, patient-centred, organisational, or interprofessional. It is conceived both as a holistic construct and as a set of appropriate and observable behaviours. Professionalism is linked to a practitioner's sense of identity, prior values, and identification with a profession, while remaining firmly grounded in the context of practice (36).
SOCIAL SCIENCES	The disciplines concerned with the interrelationships among individuals within a social environment, including social organizations and institutions. These include sociology and anthropology (37).
Bioethics and jurisprudence	- Bioethics: A branch of applied ethics that studies the value implications of practices and developments in life sciences, and health care. Bioethics includes medical ethics, which focuses on issues in healthcare; research ethics, which focuses issues on the conduct of research; environmental ethics, which focuses on issues pertaining to the relationship between human activities and the environment, and public health ethics, which addresses ethical issues in public health (38).<br>- Jurisprudence: the science or philosophy of law, and the application of legal and justice principles to health and medicine (39).
Community health and community action for health	Community health is the collective expression of the health status of individuals and groups within a community. It is determined by the interaction among individual and family characteristics, the social, cultural, and environmental context, health services, and the influence of social, political, and global factors. Community action for health encompasses all individual, collective, and intersectoral efforts directed towards improving community health (40).

*Theme is shown in uppercase letter and subtheme in lowercase letters.

Bibliography:

1. Mata ÁN de S, de Azevedo KPM, Braga LP, de Medeiros GCBS, de Oliveira Segundo VH, Bezerra INM, et al. Training in communication skills for self-efficacy of health professionals: a systematic review. Hum Resour Health. 2021 Mar 6;19(1):30.
2. NHS England. About shared decision making [Internet]. [cited 2025 Sep 1]. Available from: https://www.england.nhs.uk/personalisedcare/shared-decision-making/about/
3. Bodenheimer T, Handley MA. Goal-setting for behavior change in primary care: an exploration and status report. Patient Educ Couns. 2009 Aug;76(2):174–80.
4. World Health Organization. Health Promotion Glossary of Terms 2021 [Internet]. [cited 2025 Sep 1]. Available from: https://www.who.int/publications/i/item/9789240038349
5. Miller WR, Rollnick S. Motivational interviewing: helping people change. 3rd ed. New York: Guilford Press; 2012. 497 p.
6. NHS England. Supported self-management [Internet]. [cited 2025 Sep 1]. Available from: https://www.england.nhs.uk/personalisedcare/supported-self-management/
7. Definición de apoyo. Rasgos, tipos y herramientas [Internet]. [cited 2025 Sep 1]. Available from: https://definicion.com/apoyo/
8. Clínica Universidad de Navarra. Qué es paciente. Diccionario Médico [Internet]. [cited 2025 Sep 1]. Available from: https://www.cun.es/diccionario-medico/terminos/paciente
9. National Cancer Institute. Definition of caregiver. NCI Dictionary of Cancer Terms [Internet]. 2011 [cited 2025 Sep 1]. Available from: https://www.cancer.gov/publications/dictionaries/cancer-terms/def/caregiver

10. Roth DL, Fredman L, Haley WE. Informal caregiving and its impact on health: a reappraisal from population-based studies. Gerontologist. 2015 Apr;55(2):309–19.
11. Li J, Song Y. Formal and informal care. In: Gu D, Dupre ME, editors. Encyclopedia of Gerontology and Population Aging [Internet]. Cham: Springer; 2019 [cited 2025 Sep 1]. p. 1–8. Available from: https://link.springer.com/rwe/10.1007/978-3-319-69892-2_847-1
12. Alchemy P. Understanding the difference between clinical skills and competencies in the NHS [Internet]. People Alchemy. 2023 [cited 2025 Sep 1]. Available from: https://peoplealchemy.com/blog/difference-between-clinical-skills-and-competencies-in-the-nhs/
13. Adapted from Miller GE. The assessment of clinical skills/competence/performance. Acad Med. 1990 Sep;65(9 Suppl):S63–7.
14. Medicines management - an overview. ScienceDirect Topics [Internet]. [cited 2025 Sep 1]. Available from: https://www.sciencedirect.com/topics/nursing-and-health-professions/medicines-management
15. Oxford University Press. Challenge noun - Definition, pictures, pronunciation and usage notes. Oxford Advanced American Dictionary [Internet]. [cited 2025 Sep 1]. Available from: https://www.oxfordlearnersdictionaries.com/definition/american_english/challenge_1
16. National Cancer Institute. Definition of diagnosis. NCI Dictionary of Cancer Terms [Internet]. 2011 [cited 2025 Sep 1]. Available from: https://www.cancer.gov/publications/dictionaries/cancer-terms/def/diagnosis
17. Clínica Universidad de Navarra. Qué es tratamiento: definición médica. Diccionario Médico [Internet]. [cited 2025 Sep 1]. Available from: https://www.cun.es/diccionario-medico/terminos/tratamiento
18. European Pathway Association. Care pathways are complex interventions in complex systems: new framework [Internet]. ResearchGate; 2019 [cited 2025 Sep 1]. Available from: https://www.researchgate.net/publication/331935956
19. Ministerio de Sanidad (ES). Estrategia para el abordaje de la cronicidad [Internet]. [cited 2025 Sep 1]. Available from: https://www.sanidad.gob.es/areas/calidadAsistencial/estrategias/abordajeCronicidad/home.htm
20. GuíaSalud. Catálogo de guías de práctica clínica en el Sistema Nacional de Salud (SNS) [Internet]. [cited 2025 Sep 1]. Available from: https://portal.guiasalud.es/gpc/
21. McMaster GRADE Centre. GIN-McMaster Guideline Development Checklist [Internet]. [cited 2025 Sep 1]. Available from: https://macgrade.mcmaster.ca/resources/gin-mcmaster-guideline-development-checklist/
22. Bayot ML, Varacallo MA. Management skills. In: StatPearls [Internet]. Treasure Island (FL): StatPearls Publishing; 2025 [cited 2025 Sep 1]. Available from: http://www.ncbi.nlm.nih.gov/books/NBK544227/
23. NEJM Catalyst. What is risk management in healthcare? Catal Carryover [Internet]. 2018 Apr 25 [cited 2025 Sep 1];4(2). Available from: https://catalyst.nejm.org/doi/full/10.1056/CAT.18.0197
24. Aeon B, Faber A, Panaccio A. Does time management work? A meta-analysis. PLoS One. 2021 Jan 11;16(1):e0245066.
25. Fundación Abbott. Diccionario de gestión sanitaria para médicos: los 100 términos más utilizados. Madrid: Fundación Abbott; 2009. 331 p.
26. Cambridge University Press. Teamwork. Cambridge Dictionary [Internet]. [cited 2025 Sep 1]. Available from: https://dictionary.cambridge.org/dictionary/english/teamwork
27. Royal College of General Practitioners. Continuity of care work at RCGP [Internet]. [cited 2025 Sep 1]. Available from: https://www.rcgp.org.uk/blog/continuity-of-care-work-at-rcgp
28. Adapted from Indeed Career Guide. Work colleagues: definition and how to communicate with them [Internet]. [cited 2025 Sep 1]. Available from: https://www.indeed.com/career-advice/career-development/work-colleagues
29. American Nurses Association. ANA's Principles for Delegation by Registered Nurses to Unlicensed Assistive Personnel (UAP). Silver Spring, MD: Nursesbooks.org; 2012. 72 p. Available from: principlesofdelegation.pdf
30. McDonald KM, Sundaram V, Bravata DM, Lewis R, Lin N, Kraft SA, et al. Definitions of care coordination and related terms. In: Closing the Quality Gap: A Critical Analysis of Quality Improvement Strategies (Vol 7: Care Coordination) [Internet]. Rockville (MD): Agency for Healthcare Research and Quality (US); 2007 [cited 2025 Sep 1]. Available from: https://www.ncbi.nlm.nih.gov/books/NBK44012/
31. GP Website. Referral management [Internet]. [cited 2025 Sep 1]. Available from: https://gps.cityandhackneyccg.nhs.uk/topic/referral-management
32. CASP - Critical Appraisal Skills Programme. What is critical appraisal? [Internet]. [cited 2025 Sep 1]. Available from: https://casp-uk.net/what-is-critical-appraisal/
33. European Skills, Competences, Qualifications and Occupations (ESCO). Competence [Internet]. [cited 2025 Apr 16]. Available from: https://esco.ec.europa.eu/en/about-esco/escopedia/escopedia/competence
34. California State University San Marcos. ICT and exclusions defined. Instructional and Information Technology Services (IITS) [Internet]. [cited 2025 Sep 1]. Available from: https://www.csusm.edu/iits/itr-process/ict_and_exclusions_defined.html
35. Cao H, Song Y, Wu Y, Du Y, He X, Chen Y, et al. What is nursing professionalism? A concept analysis. BMC Nurs. 2023 Feb 7;22(1):34.
36. Adapted from Morrow G, Burford B, Rothwell C, Carter M, McLachlan J, Illing J. Professionalism in healthcare professionals. London: Health and Care Professions Council; 2014. 51 p. ISBN: 978-1-910938-02-7.
Available from: professionalism-in-healthcare-professionals.pdf
37. National Center for Biotechnology Information. Social sciences - MeSH [Internet]. [cited 2025 Sep 1]. Available from: https://www.ncbi.nlm.nih.gov/mesh/68012942
38. National Center for Biotechnology Information. Bioethics - MeSH [Internet]. [cited 2025 Sep 1]. Available from: https://www.ncbi.nlm.nih.gov/mesh/68001675
39. National Center for Biotechnology Information. Jurisprudence - MeSH [Internet]. [cited 2025 Sep 1]. Available from: https://www.ncbi.nlm.nih.gov/mesh/68007603
40. Fernández G. Salud comunitaria basada en activos. Escuela Andaluza de Salud Pública [Internet]. 2019 [cited 2025 Sep 1]. Available from: https://www.easp.es/project/salud-comunitaria-basada-en-activos/

Additional file 6:

TABLE AF6. Themes, subthemes and codes

Themes	Subthemes	Codes
COMPETENCIES IN COMMUNICATION	General	C2- Communication<br>C27- Active listening<br>C91- Managing bad news
	Joint decision-making and goal setting	C31- Shared decision-making<br>C111- Patient-centered care<br>C127- Prioritizing the patient's needs<br>C156- Individualization
	Lifestyle advice	C48- Other ways to take care of yourself<br>C52- Health education
	Motivational interviewing	C7- Motivational interviewing<br>C108- Patient motivation strategies
	Instruction in self-management	C22- Self-care<br>C25- Nurse education
	Patient and carer support	C62- Carers (training, care)<br>C155- Mourning<br>C161- Family-centered care<br>C184- Improve the relationship with the patient/family
CLINICAL COMPETENCIES	General	C10- Clinical skills
	Medicines management	C1- Polypharmacy<br>C4- Drugs interaction<br>C5- Evidence-based drugs prescription<br>C8- Deprescription<br>C12- Kidney failure and treatments<br>C16- Anticholinergic burden<br>C26- Therapeutic adherence<br>C38- Drugs side effects<br>C39- Antibiotic resistance<br>C46- Rational use of medicines<br>C53- Discontinued treatments<br>C68- Liver diseases and treatments<br>C70- Interactions with osteopathic treatments<br>C88- Drug combinations<br>C89- Therapeutic innovations<br>C93- Psychotropic drugs<br>C96- Treatment conciliation<br>C103- Most prevalent treatments<br>C107- Drugs and dehydration<br>C109- Treatment prioritization<br>C128- Disease-drug interactions<br>C146- Diet and treatments
	Diagnostic and treatment challenges	C11- Comorbidities<br>C15- Chronic pain treatment<br>C18- Pharmacotherapy<br>C21- Measuring tools<br>C23- Treatment adequacy<br>C24- Non-pharmacological therapies<br>C33- Nutritional care<br>C35- Disease prioritization<br>C36- Comprehensive geriatric assessment<br>C40- Diagnostic peculiarities<br>C41- Geriatric syndromes<br>C42- Medicinal plants<br>C44- Home management of acute problems<br>C51- Update on chronic diseases<br>C55- COPD<br>C56- Diabetes<br>C57- Hypertension<br>C60- Arrhythmias<br>C61- Cognitive assessment<br>C63- Handling emergency situations<br>C64- General care<br>C65- Addictions<br>C66- Mental health<br>C69- Cardiovascular diseases<br>C71- Comprehensive treatment of cardiovascular risk factors<br>C73- Quality of life<br>C74- Comprehensive approach

		C75- Limitation of therapeutic effort<br>C77- Pressure ulcers<br>C80- Attention to the most common diseases<br>C83- Disease follow-up<br>C84- Problem solving approach<br>C86- Vital prognosis<br>C87- Warning signs<br>C90- Pregnancy and breastfeeding<br>C92- Management of decompensations<br>C94- Heart failure<br>C97- Ultrasound<br>C98- Clinical care for multiple symptoms<br>C104- Nursing care<br>C105- Psychological approach<br>C106- End-of-life care<br>C110- Life expectancy<br>C116- Obesity<br>C117- Therapeutic objectives<br>C118- Psychological Therapies<br>C121- Euthanasia<br>C122- Symptomatic treatment<br>C124- Assessment scales<br>C125- Immunosuppression<br>C126- Paediatrics<br>C130- Actions in cancer patients<br>C131- Metabolic syndrome<br>C132- Enolism<br>C133- ECG for nurses<br>C140- Insomnia Management<br>C141- Agitation management<br>C142- Stratification (risk, etc.)<br>C143- Dyslipidaemia<br>C144- Home care<br>C145- Disease-disease Interactions<br>C147- Admission prevention<br>C148- Most prevalent diseases<br>C152- Exacerbations of diseases<br>C153- Wounds<br>C157- Neurological diseases<br>C159- Dyspnoea<br>C162- Digestive disease<br>C171- Difficulty in interpreting tests<br>C187- Clinical experience
	Complex care pathways	C6- Management of patients with multiple diseases (serious, safe drugs, etc.)<br>C13- Actions in patients with dementia<br>C14- Palliative care<br>C28- Actions in patients with multimorbidity<br>C34- Frailty care<br>C95- Comprehensive care for complex patients
	Application and adaptation of guidelines	C17- Clinical practice guidelines<br>C19- Protocols
MANAGEMENT COMPETENCIES	General	
	Risk management	C30- Prevention in elderly patients<br>C72- Patient safety<br>C85- Prescribing cascade<br>C100- Overdiagnosis<br>C102- Quaternary prevention<br>C112- Do not do recommendations<br>C120- Handling in risky situations<br>C123- Overtreatment<br>C136- Therapeutic inertia
	Time management	C20- Management (time, etc.)<br>C163- Lack of time<br>C190- Time
	Clinical management	C54- Efficiency<br>C67- Care in pandemic<br>C78- Accessibility<br>C79- Health and non-health resources<br>C82- Primary care<br>C84- Problem solving approach<br>C99- Scheduled and on-demand consultation<br>C113- Work in stages<br>C158- Economic analysis<br>C164- Lack of space

		C166- Lack of institutional support for professionals<br>C168- Inadequate care organization<br>C169- Barriers to applying the characteristics of primary care<br>C170- Insufficient resources (computing, etc.)<br>C172- High-pressure healthcare settings<br>C173- Architectural barriers<br>C174- Different types of care (in person, by phone, etc.)<br>C175- Lack of professionals<br>C176- Work problems (contracts, etc.)<br>C177- Less frequentation of patients<br>C179- Difficulty in reconciling the professional's personal life<br>C180- Variability in clinical practice<br>C182- Organizational improvements in care<br>C183- Longitudinal<br>C188- Active attracting with patients<br>C193- Low-pressure healthcare
TEAMWORK COMPETENCIES	General	C9- Teamwork *
	Continuity of care	C119- Family medicine<br>C151- Transition to discharge<br>C160- Continuity of care
	Working with colleagues	C9- Teamwork *<br>C43- Skills to motivate professionals *
	Delegation and coordination of care	C45- Relationship between levels of care<br>C49- Public health coordination<br>C129- Rotations by coordination units<br>C151- Transition to discharge
	Referral management	C45- Relationship between levels of care<br>C135- Referrals
CRITICAL APPRAISAL COMPETENCIES		C5- Evidence-based drug indications<br>C50- Evaluation<br>C59- Critical appraisal<br>C81- Clinical cases
COMPETENCIES IN INFORMATION TECHNOLOGY		C58- ICTs (for clinical monitoring, etc.)<br>C138- Information systems
PROFESSIONALISM		C43- Skills to motivate professionals<br>C101- Prescription and relationship of the professional with the pharmaceutical industry<br>C114- Training<br>C137- Research<br>C150- Humanization<br>C165- Lack of professional training<br>C178- Lack of motivation in professionals<br>C181- Self-learning (information search, etc.)<br>C185- Professional with positive attitudes/emotions towards care<br>C186- Continuing training for professionals<br>C189- Professionalism<br>C191- Teaching role of the professional<br>C192- Specialized health training
COMPETENCIES IN SOCIAL SCIENCES	General	C49- Public health coordination
	Bioethics and jurisprudence	C76- Legal rights<br>C154- Ethics
	Community health and community action for health	C32- Community care<br>C37- Dependency care (social resources, etc.)<br>C47- Psychosocial support<br>C115- Social assessment<br>C134- Elimination of sociocultural barriers<br>C139- Local resources<br>C149- Socioeconomic determinants<br>C167- Population aging<br>C194- Rural area

* Reclassified according to the response

COPD: Chronic obstructive pulmonary disease; ECG: Electrocardiogram; ICTs: Information and communications technologies

Additional file 7:

TABLE AF7. Comparison between the national primary care workforce in Spain (2022) and the study sample

Characteristic	National workforce** n (%)	Study sample n (%)
**Profession**		
Doctors (including residents)	45.083 (53.9)	270 (70.1)
Nurses (including residents)	38.554 (46.1)	115 (29.9)
**Training status**		
Non-resident professionals	73.744 (88.2)	353 (91.7)
Residents (total)	9.893 (11.8)	32 (8.3)
**Current professional role***		
Primary Care Team	59.633 (80.9)	284 (73.8)
Out-of-hours/emergency services	14.111 (19.1)	21 (5.5)
Both	—	61 (15.8)

Notes:

Women represent approximately 69.8% of physicians and 71.9% of nurses in the national workforce, compared with 70.7% and 81.7%, respectively, in the study sample.

*Residents were excluded from the national distribution of professional setting because their training includes rotations across different healthcare settings.

**Data from Ministerio de Sanidad. Recursos Humanos, ordenación profesional y formación continuada en el Sistema Nacional de Salud, 2022. Informe monográfico. Madrid: Centro de Publicaciones; 2023. Disponible en: https://cpage.mpr.gob.es/

Additional file 8:

TABLE AF8. Characteristics of study participants (n = 385)

**Age** mean [SD]	45.9 [12.04]		
**Gender** n (%)	Male		104 (27)
	Female		280 (72.7)
	Other (fluid)		1 (0.3)
**Survey distribution channel** n (%)	Corporate email		138 (35.8)
	Personal email		72 (18.7)
	Social media (Twitter, Facebook, Instagram, LinkedIn…)		81 (21)
	Instant messaging (WhatsApp, Telegram, Messenger, Line…)		94 (24.4)
**Province*** n (%)	Most frequent provinces	Málaga	73 (19)
		Madrid	29 (7.5)
		Cantabria	27 (7)
		Burgos	21 (5.5)
		Barcelona	19 (4.9)
**Current profession** n (%)	Doctor		254 (66)
	Nurse		99 (25.7)
	Family and Community Medicine resident		16 (4.2)
	Family and Community Nursing resident		16 (4.2)
**Current professional role** n (%)	Primary Care Team		284 (73.8)
	Primary Care Emergency Service/Out-of-hospital emergencies		21 (5.5)
	Both		61 (15.8)
	Other (management, private service, nursing home, palliative care)		19 (4.9)
**Work setting** n (%)	Urban		227 (59)
	Rural		113 (29.4)
	Both		43 (11.2)
	Others (management)		2 (0.5)
**Participation in MULTIPAP** n (%)	Yes		37 (9.6)
	No		348 (90.4)
**Previous training in MM** n (%)	Yes		274 (71.2)
	No		111 (28.8)
**Type of MM training received** n (%)	Face-to-face course		81 (21)
	Online course		160 (41.6)
	Single talk or seminar		74 (19.2)
	Self-training		113 (29.4)
**Timing of MM training** n (%)	Last year		72 (18.7)
	1–5 years ago		150 (39)
	More than 5 years ago		46 (11.9)
**Length of MM training** n (%)	Less than 30 hours		179 (46.5)
	30 hours or more		94 (24.4)
**MM training content** n (%)	Communication skills		106 (27.5)
	Clinical skills		238 (61.8)
	Management skills		66 (17.1)
	Critical appraisal skills		68 (17.7)
	Teamwork		71 (18.4)
	Information Technology skills		31 (8.1)

MM: multimorbidity; SD: standard deviation

*Responses were obtained from 45 of the 52 Spanish provinces or autonomous cities. Full provincial distribution is provided in Supplementary Figure AF9

Additional file 9:

FIGURE AF9. Geographical distribution of study participants by province (%)

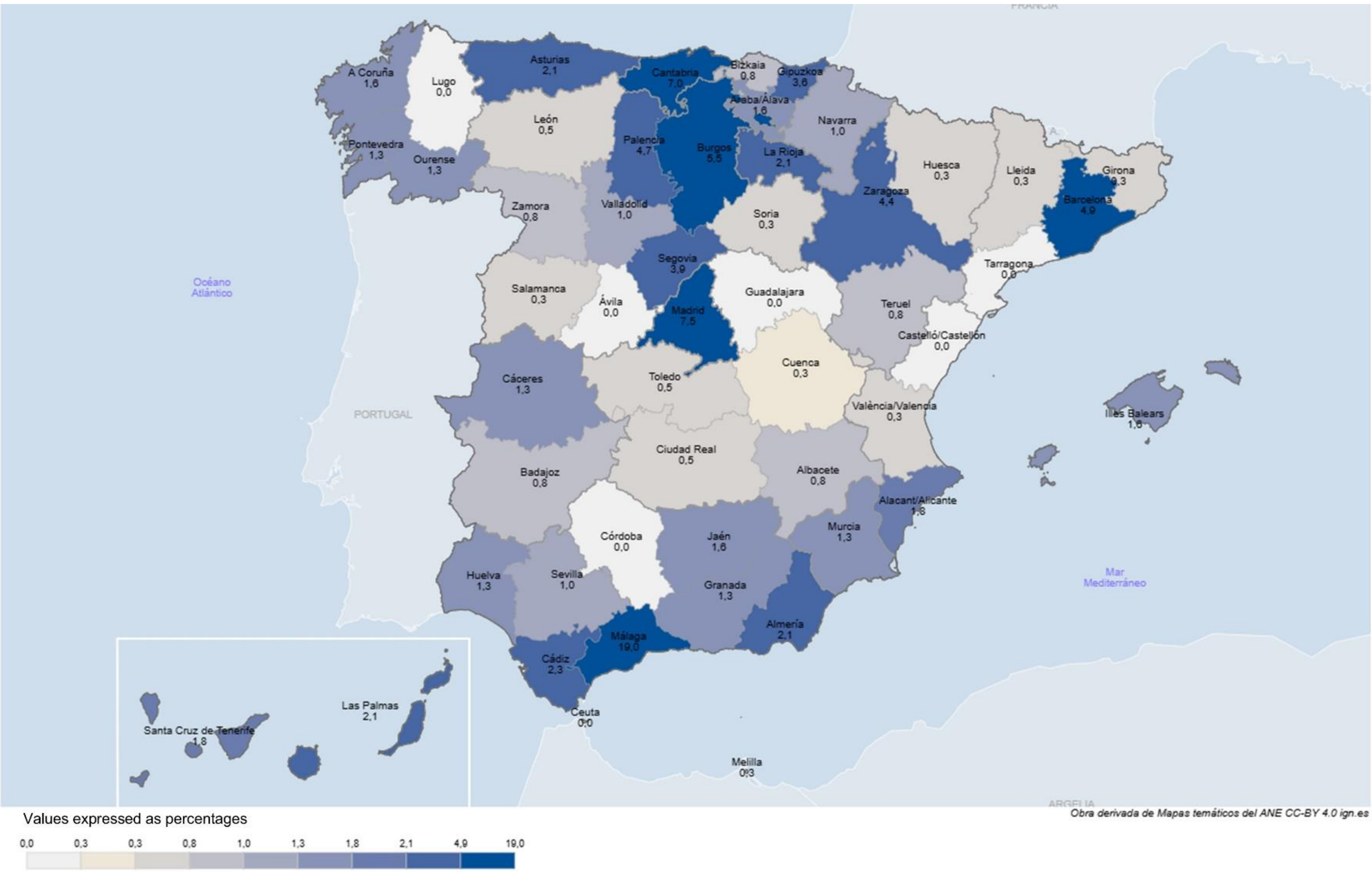

Additional file 10:

TABLE AF10: Comparison of perceived training needs, training priorities, difficulties and tools according to the operational definition of multimorbidity used by respondents.

Domain	Competency	EGPRN (n=203) n (%)	≥3 conditions threshold (n=77) n (%)	≥2 conditions (WHO) (n=102) n (%)	p-value
**Training needs**	Communication competencies	62 (30.8)	29 (37.7)	32 (32.7)	0.556
	Clinical skills	186 (92.5)	74 (96.1)	95 (96.9)	0.229
	Information technology skills	13 (6.4)	5 (6.5)	6 (5.9)	0.981
	Teamwork	25 (12.3)	10 (13.0)	16 (15.7)	0.713
	Critical appraisal skills	12 (5.9)	5 (6.5)	7 (6.9)	0.948
	Management skills	47 (23.2)	9 (11.7)	16 (15.7)	0.058
	Professionalism	8 (4.0)	3 (3.9)	3 (3.1)	0.922
	Social sciences	12 (6.0)	5 (6.5)	6 (6.1)	0.987
**Training priorities**	Communication competencies	29 (15.2)	11 (14.7)	13 (13.5)	0.933
	Clinical skills	149 (78.0)	67 (89.3)	86 (89.6)	**0.014**
	Information technology skills	1 (0.5)	0 (0.0)	1 (1.0)	0.664
	Teamwork	11 (5.4)	5 (6.5)	5 (4.9)	0.896
	Critical appraisal skills	7 (3.4)	4 (5.2)	1 (1.0)	0.260
	Management skills	31 (15.3)	7 (9.1)	11 (10.8)	0.297
	Professionalism	1 (0.5)	1 (1.3)	0 (0.0)	0.504
	Social sciences	6 (3.1)	1 (1.3)	4 (4.2)	0.559
**Difficulties**	Communication competencies	28 (13.9)	15 (19.7)	16 (16.3)	0.487
	Clinical skills	85 (42.3)	41 (53.9)	50 (51.0)	0.142
	Information technology skills	2 (1.0)	0 (0.0)	0 (0.0)	0.412
	Teamwork	29 (14.3)	17 (22.1)	22 (21.6)	0.160
	Critical appraisal skills	2 (1.0)	0 (0.0)	1 (1.0)	0.683
	Management skills	125 (61.6)	45 (58.4)	51 (50.0)	0.154
	Professionalism	27 (13.4)	4 (5.3)	10 (10.2)	0.146
	Social sciences	12 (6.0)	4 (5.3)	6 (6.1)	0.968
**Tools**	Communication competencies	32 (16.4)	13 (17.6)	25 (26.9)	0.100
	Clinical skills	56 (28.7)	27 (36.5)	30 (32.3)	0.456
	Information technology skills	21 (10.3)	8 (10.4)	11 (10.8)	0.993
	Teamwork	51 (25.1)	21 (27.3)	27 (26.5)	0.925
	Critical appraisal skills	4 (2.0)	0 (0.0)	1 (1.0)	0.408
	Management skills	47 (23.2)	21 (27.3)	17 (16.7)	0.217
	Professionalism	91 (46.7)	30 (40.5)	43 (46.2)	0.652
	Social sciences	6 (3.1)	1 (1.4)	1 (1.1)	0.476

EGPRN: European General Practice Research Network; WHO: World Health Organization

Values are presented as number (percentage). Differences between groups were assessed using chi-square tests. Some comparisons included small cell counts and should therefore be interpreted with caution.

Additional file 11:

TABLE AF11. Likert-scale data. Importance attributed to a series of predefined competencies*

	1 (no.)	2 (no.)	3 (no.)	4 (no.)	TOTAL (n)	Mean	SD	Median	IQR	Mode
Patient and carer support	1	5	29	350	385	3.89	0.373	4	0	4
Medicines management	1	5	76	303	385	3.77	0.469	4	0	4
Patient-centered care	0	13	61	311	385	3.77	0.493	4	0	4
Continuity and coordination of care	2	11	75	297	385	3.73	0.534	4	0	4
Joint decision-making	3	14	69	299	385	3.72	0.566	4	0	4
Lifestyle advice and instruction in self-management	1	18	85	281	385	3.68	0.573	4	1	4
Time management	3	33	120	229	385	3.49	0.685	4	1	4
Risk management	1	25	151	208	385	3.47	0.629	4	1	4
Development of care plans	4	45	131	205	385	3.39	0.732	4	1	4
Application and adaptation of guidelines	3	43	167	172	385	3.32	0.699	3	1	4
Electronic health records and information collation	5	54	196	130	385	3.17	0.708	3	1	3
Critical appraisal skills and problem solving	3	55	199	128	385	3.17	0.691	3	1	3
Sum	27	321	1359	2913						

*Combination of original data and data adapted from Lewis C. Wallace E. Kyne L. Cullen W. Smith SM. Training doctors to manage patients with multimorbidity: a systematic review. J Comorb. 2016 Aug 26;6(2):85-94. doi: 10.15256/joc.2016.6.87. PMID: 29090179; PMCID: PMC5556450.

SD: Standard deviation; IQR: Interquartile range

Additional file 12:

TABLE AF12. Themes and subthemes distribution

Themes	n (%)	Subthemes	n (%)
**TRAINING NEEDS**			
**Themes**	**n (%)**	**Subthemes**	**n (%)**
COMPETENCIES IN COMMUNICATION	129 (32.3)	Communication competence (general)	80 (20.1)
		Joint decision-making and goal setting	18 (4.5)
		Lifestyle advice	23 (5.8)
		Motivational interviewing	7 (1.8)
		Instruction in self-management	6 (1.5)
		Patient and carer support	10 (2.5)
CLINICAL COMPETENCIES	377 (94.5)	Clinical skills (general)	40 (10)
		Medicines management	254 (63.7)
		Diagnostic and treatment challenges	238 (59.6)
		Complex care pathways	108 (27.1)
		Application and adaptation of guidelines	17 (4.3)
MANAGEMENT COMPETENCIES	25 (6.3)		
TEAMWORK COMPETENCIES	54 (13.5)	Teamwork (general)	35 (8.8)
		Continuity of care	3 (0.8)
		Working with colleagues	0
		Delegation and coordination of care	18 (4.5)
		Referral management	14 (3.5)
CRITICAL APPRAISAL COMPETENCIES	26 (6.5)		
COMPETENCIES IN INFORMATION TECHNOLOGY	75 (18.8)	Risk management	27 (6.8)
		Time management	37 (9.3)
		Clinical management	13 (3.3)
PROFESSIONALISM	16 (4)		
COMPETENCIES IN SOCIAL SCIENCES	28 (7)	Social sciences (general)	3 (0.8)
		Bioethics and jurisprudence	3 (0.8)
		Community health and community action for health	23 (5.8)
**TRAINING PRIORITIES**			
**Themes**	**n (%)**	**Subthemes**	**n (%)**
COMPETENCIES IN COMMUNICATION	58 (15)	Communication skills (general)	30 (7.8)
		Joint decision-making and goal setting	9 (2.3)
		Lifestyle advice	15 (3.9)
		Motivational interviewing	2 (0.5)
		Instruction in self-management	4 (1)
		Patient and carer support	6 (1.6)
CLINICAL COMPETENCIES	323 (83.7)	Clinical skills (general)	25 (6.5)
		Medicines management	124 (32.1)
		Diagnostic and treatment challenges	165 (42.7)
		Complex care pathways	103 (26.7)
		Application and adaptation of guidelines	9 (2.3)
MANAGEMENT COMPETENCIES	2 (0.5)		
TEAMWORK COMPETENCIES	22 (5.7)	Teamwork (general)	13 (3.4)
		Continuity of care	2 (0.5)
		Working with colleagues	2 (0.5)
		Delegation and coordination of care	8 (2.1)
		Referral management	8 (2.1)
CRITICAL APPRAISAL COMPETENCIES	12 (3.1)		
COMPETENCIES IN INFORMATION TECHNOLOGY	51 (13.2)	Risk management	16 (4.1)
		Time management	22 (5.7)
		Clinical management	15 (3.9)
PROFESSIONALISM	3 (0.8)		
COMPETENCIES IN SOCIAL SCIENCES	12 (3.1)	Social sciences (general)	7 (1.8)
		Bioethics and jurisprudence	1 (0.3)
		Community health and community action for health	4 (1)
**DIFFICULTIES**			
**Themes**	**n (%)**	**Subthemes**	**n (%)**
COMPETENCIES IN COMMUNICATION	60 (14.9)	Communication competence (general)	11 (2.7)
		Joint decision-making and goal setting	29 (7.2)
		Lifestyle advice	9 (2.2)
		Motivational interviewing	3 (0.7)

Themes	n (%)	Subthemes	n (%)
		Instruction in self-management	10 (2.5)
		Patient and carer support	9 (2.2)
CLINICAL COMPETENCIES	184 (45.8)	Clinical skills (general)	1 (0.2)
		Medicines management	90 (22.4)
		Diagnostic and treatment challenges	84 (20.9)
		Complex care pathways	55 (13.7)
		Application and adaptation of guidelines	9 (2.2)
MANAGEMENT COMPETENCIES	2 (0.5)		
TEAMWORK COMPETENCIES	74 (18.4)	Teamwork (general)	11 (2.7)
		Continuity of care	0
		Working with colleagues	14 (3.5)
		Delegation and coordination of care	63 (15.7)
		Referral management	57 (14.2)
CRITICAL APPRAISAL COMPETENCIES	3 (0.7)		
COMPETENCIES IN INFORMATION TECHNOLOGY	241 (60)	Risk management	14 (3.5)
		Time management	183 (45.5)
		Clinical management	114 (28.4)
PROFESSIONALISM	43 (10.7)		
COMPETENCIES IN SOCIAL SCIENCES	22 (5.5)	Social sciences (general)	0
		Bioethics and jurisprudence	0
		Community health and community action for health	22 (5.5)
**TOOLS**			
**Themes**	**n (%)**	**Subthemes**	**n (%)**
COMPETENCIES IN COMMUNICATION	75 (19.6)	Communication skills (general)	48 (12.6)
		Joint decision-making and goal setting	15 (3.9)
		Lifestyle advice	8 (2.1)
		Motivational interviewing	2 (0.5)
		Instruction in self-management	0
		Patient and carer support	11 (2.9)
CLINICAL COMPETENCIES	117 (30.6)	Clinical skills (general)	8 (2.1)
		Medicines management	7 (1.8)
		Diagnostic and treatment challenges	65 (17)
		Complex care pathways	5 (1.3)
		Application and adaptation of guidelines	43 (11.3)
MANAGEMENT COMPETENCIES	43 (11.3)		
TEAMWORK COMPETENCIES	104 (27.2)	Teamwork (general)	0
		Continuity of care	5 (1.3)
		Working with colleagues	85 (22.3)
		Delegation and coordination of care	21 (5.5)
		Referral management	21 (5.5)
CRITICAL APPRAISAL COMPETENCIES	6 (1.6)		
COMPETENCIES IN INFORMATION TECHNOLOGY	88 (23)	Risk management	2 (0.5)
		Time management	24 (6.3)
		Clinical management	64 (16.8)
PROFESSIONALISM	172 (45)		
COMPETENCIES IN SOCIAL SCIENCES	5 (1.3)	Social sciences (general)	0
		Bioethics and jurisprudence	0
		Community health and community action for health	5 (1.3)

Additional file 13:

TABLE AF13. Verbatim responses by theme and subtheme on difficulties and tools in multimorbidity management

Themes	Subthemes	E.g. answers about difficulties	E.g. answers about tools
COMPETENCIES IN COMMUNICATION	General	*Communication difficulties caused by sensory problems.*	*Communication is the fundamental pillar for explaining to the patient their situation and being able to work with them.*
	Joint decision-making and goal setting	*To coordinate patients' best interests and needs with their drug cocktails. To convince a patient to take their 16 daily pills when they ask if we could remove some.*	*Individualize and prioritize and focus on the patient's needs.*
	Lifestyle advice	*Getting my patients to adopt lifestyle changes that have a real impact on their chronic conditions.*	*Time to dedicate to these patients, with educational materials designed just for them.*
	Motivational interviewing	*Disease awareness.*	*Motivational interviewing.*
	Instruction in self-management	*To train the patient to detect decompensations and improve self-care.*	
	Patient and carer support	*Encouraging families to get involved and make use of the support available can be challenging.*	*Connection with the patient and family.*
CLINICAL COMPETENCIES	General	*To carry out basic life support techniques.*	*Clinical skills and understanding patients in various spheres.*
	Medicines management	*Identifying interactions, gaps and treatment management during consultations in a complex context, which makes the efficient and safe management of medications more challenging.*	*Medication reconciliation, after hospital discharges or periodically following exacerbations. On discharge, patients usually return with the treatment for the acute episode, with the rest of their medication "unchanged". Occasionally, for example with a diuretic, they may already be taking one and are prescribed an additional one.*
	Diagnostic and treatment challenges	*Management of decompensations of chronic pathologies.*	*Regular follow-ups in clinic, either face-to-face or over the phone.*
	Complex care pathways	*Many healthcare professionals treat patients in a fragmented way rather than taking a holistic approach. There should be a dedicated unit for patients with multiple chronic conditions. Even within Internal Medicine, which manages a wide range of pathologies, there are many specialties, and each one provides treatment for its own area.*	*Referral to a specialised clinic for patients with multiple chronic conditions.*
	Application and adaptation of guidelines	*Lack of clinical practice guidelines that take into account complexity, which gives the impression of "inventing" solutions.*	*Clinical practice guidelines, although they are independent and do not take multimorbidity into account.*
MANAGEMENT COMPETENCIES	Risk management	*The integration of pathologies and their severity and choosing the safest treatments.*	*Careful management, minimal intervention.*
	Time management	*The lack of time to address everything, recognising that multimorbidity is not only influenced by purely medical factors, and that while we identify social determinants in consultation, we are unable to address them.*	*Manage my own consultation time in the schedule.*
	Clinical management	*Waiting times and distance from specialist care.*	*Trying to schedule appointments on the same day with different temporary staff to reduce absenteeism, and arranging*

			additional appointments for assessments, etc.
TEAMWORK COMPETENCIES	Continuity of care	*Difficulty accessing care and discontinuity of care due to frequently changing primary care teams (short-term contracts).*	*Coordinating the primary care team to ensure continuity of care.*
	Working with colleagues	*Reduced time for collaborative work between doctors and nurses.*	*Working as part of a team with nurses, social workers and pharmacists.*
	Delegation and coordination of care	*The limited or absent real communication with other specialist colleagues makes it difficult to make shared decisions between professionals, patients, and their families.*	*Coordination with the hospital's liaison nurses and the local authority's social services.*
	Referral management	*Consolidating all instructions and decisions from each specialist to avoid repeating tests and overmedication (iatrogenesis).*	*Teleconsultation with hospital-based professionals.*
CRITICAL APPRAISAL COMPETENCIES		*Much of the available evidence is produced by private companies (the WHO, for instance, is around 80% privately funded).*<br>*Limited time and resources to critically appraise articles.*<br>*Lack of time and opportunities to engage in meaningful scientific debate.*<br>*Media neuromarketing that shapes how evidence is presented, often influenced by commercial rather than clinical interests.*	*Concise bibliographic reviews without conflicts of interest.*
INFORMATION TECHNOLOGY COMPETENCE		*Lack of ICT support to better assess drug interactions in polymedicated patients.*	*IT tools to identify patients with multimorbidity.*
PROFESSIONALISM		*Lack of training opportunities for professionals within their workplaces and during working hours.*	*Patience, self-directed learning, and dedication beyond working hours.*
COMPETENCIES IN SOCIAL SCIENCES	General		*Home visits.*<br>*Social support services.*
	Bioethics and jurisprudence		
	Community health and community action for health	*They live alone or with a partner in a similar situation, in an environment with few opportunities for social support or neighbourly contact, and face difficulties accessing the Health Centre or hospital due to a lack of transport.*	*Community work to gain a better understanding of the patient's environment.*

E.g.: for example
IT: Information Technology; ICT: Information and Communication Technology

Additional file 14:

TABLE AF14. Bivariate analysis results of significant associations for "Training needs"

Theme/Subtheme	Factor	n (%) or Mean age ± SD	P-value
Lifestyle advice	Male	2 (2.0)	0.051 [1]
	Female	20 (7.2)	
	Doctor	10 (3.7)	0.008 [1]
	Nurse	12 (10.7)	
	Age (years)	40.1±12.7 vs 46.4±11.8 [4]	0.016 [3]
Instruction in self-management	Male	4 (3.8)	0.048 [2]
	Female	2 (0.7)	
	Doctor	0 (0.0)	0.001 [2]
	Nurse	6 (5.2)	
Medicines management	Male	73 (71.6)	0.022 [1]
	Female	162(58.7)	
	Doctor	179 (67.0)	0.003 [1]
	Nurse	57 (50.9)	
Joint decision-making and goal setting	Doctor	16 (6.0)	0.029 [1]
	Nurse	1 (0.9)	
	Participate in the MULTIPAP Study	6 (16.2)	0.003 [2]
	Non-participation in the MULTIPAP Study	11 (3.2)	
	Age (years)	52.4±12.3 vs 45.7±11.9 [4]	0.024 [3]
Competencies in information technology	Urban	20 (8.8)	0.033 [1]
	Rural	3 (2.7)	
	Age (years)	50.8±10.0 vs 45.6±12.1 [4]	0.019 [3]
Competencies in communication (general)	Participate in the MULTIPAP Study	13 (35.1)	0.015 [1]
	Non-participation in the MULTIPAP Study	64 (18,4)	

[1] Chi-square test; [2] Fisher's exact test; [3] Student's t-test; [4] Mean age ±SD of respondents reporting training needs versus those not reporting them

Additional file 15:

TABLE AF15. Bivariate analysis results of significant associations for "Training priorities"

Theme/Subtheme	Factor	n (%) or Mean age ± SD	P-value
Competencies in communication	Doctor	29 (11.3)	0.008 [1]
	Nurse	24 (22.0)	
Instruction in self-management	Doctor	0 (0.0)	0.008 [1]
	Nurse	4 (3.5)	
Medicines management	Doctor	101 (39.5)	0.000 [1]
	Nurse	16 (14.7)	
	Primary care team	96 (35.4)	0.040 [1]
	Primary Care Emergency Service+out-of-hospital emergencies+Both of them+Others	18 (23.1)	
Clinical competencies	Resident	29 (96.7)	0.041 [2]
	Non-resident	276 (82.4)	
Complex care pathways	Urban	46 (21.3)	0.022 [1]
	Rural	36 (33.0)	
Competencies in social sciences	Participate in the MULTIPAP Study	3 (8.8)	0.073 [2]
	Non-participation in the MULTIPAP Study	8 (2.4)	
Joint decision-making and goal setting	Participate in the MULTIPAP Study	3 (8.8)	0.042 [1]
	Non-participation in the MULTIPAP Study	6 (1.8)	
Competencies in social sciences (general)	Participate in the MULTIPAP Study	2 (5.4)	0.105 [2]
	Non-participation in the MULTIPAP Study	4 (1.1)	
Lifestyle advice	Age (years)	37.8±10.3 vs 46.1±12.0 [4]	0.014 [3]

[1] Chi-square test; [2] Fisher's exact test; [3] Student's t-test; [4] Mean age ±SD of respondents reporting training priorities versus those not reporting them

Additional file 16:

TABLE AF16. Bivariate analysis results of significant associations for "Difficulties"

Theme/Subtheme	Factor	n (%) or Mean age ± SD	P-value
Management competencies	Male	37 (35.6)	0.033 [1]
	Female	69 (24.6)	
Competencies in social sciences	Doctor	10 (3.7)	0.008 [1]
	Nurse	12 (10.8)	
	Rural	12 (10.8)	0.008 [1]
	Urban	8 (3.6)	
Joint decision-making and goal setting	Doctor	26 (9.7)	0.019 [1]
	Nurse	3 (2.7)	
	Participation in the MULTIPAP Study	7 (19.4)	0.013 [2]
	Non-participation in the MULTIPAP Study	22 (6.4)	
Lifestyle advice	Doctor	2 (0.7)	0.009 [2]
	Nurse	6 (5.4)	
Competencies in communication (general)	Doctor	4 (1.5)	0.02 [2]
	Nurse	7 (6.1)	
Medicines management	Doctor	70 ((26.2)	0.013 [1]
	Nurse	16 (14.4)	
Complex care pathways	Doctor	31 (11.6)	0.021 [1]
	Nurse	23 (20.7)	
Time management	Doctor	135 (50.0)	0.000 [1]
	Nurse	33 (28.7)	
	Only primary care team	132 (46.5)	0.047 [1]
	Primary Care Emergency Service+out-of-hospital emergencies+Both of them+Others	28 (34.1)	
Community health and community action for health	Doctor	10 (3.7)	0.009 [1]
	Nurse	12 (10.4)	
	Rural	12 (10.6)	0.009 [1]
	Urban	8 (3.5)	
Clinical competencies	Rural	64 (57.7)	0.007 [1]
	Urban	94 (42.0)	
Instruction in self-management	Rural	8 (7.1)	0.001 [2]
	Urban	1 (0.4)	
Teamwork competencies	Only primary care team	59 (20.8)	0.005 [1]
	Primary Care Emergency Service+out-of-hospital emergencies+Both of them+Others	6 (7.3)	
	Participation in the MULTIPAP Study	12 (32.4)	0.015 [1]
	Non-participation in the MULTIPAP Study	57 (16.4)	
	Age (years)	48.6±11.0 vs 45.3±12.2 [4]	0.038 [3]
Motivational interviewing	Only primary care team	0 (0.0)	0.050 [2]
	Primary Care Emergency Service+out-of-hospital emergencies+Both of them+Others	2 (2.4)	
Working with colleagues	Only primary care team	14 (4.9)	0.046 [2]
	Primary Care Emergency Service+out-of-hospital emergencies+Both of them+Others	0 (0.0)	
Delegation and coordination of care	Only primary care team	48 (16.9)	0.031 [1]
	Primary Care Emergency Service+out-of-hospital emergencies+Both of them+Others	6 (7.3)	
	Participation in the MULTIPAP Study	12 (32.4)	0.002 [1]
	Non-participation in the MULTIPAP Study	46 (13.2)	
	Age (years)	48.8±10.9 vs 45.4±12.2 [4]	0.035 [3]
Referral management	Only primary care team	44 (15.5)	0.028 [1]
	Primary Care Emergency Service+out-of-hospital emergencies+Both of them+Others	5 (6.1)	
	Participation in the MULTIPAP Study	11 (29.7)	0.009 [1]
	Non-participation in the MULTIPAP Study	41 (11.8)	
Competencies in communication	Participation in the MULTIPAP Study	10 (27.8)	0.034 [1]
	Non-participation in the MULTIPAP Study	49 (14.3)	
Application and adaptation of guidelines	Participation in the MULTIPAP Study	4 (11.1)	.004 [1]
	Non-participation in the MULTIPAP Study	4 (1.2)	
	Age (years)	37.1±6.7 vs 46.2±12.0 [4]	0.006 [3]

[1] Chi-square test; [2] Fisher's exact test; [3] Student's t-test; [4] Mean age ±SD of respondents reporting difficulties versus those not reporting them

Additional file 17:

TABLE AF17. Bivariate analysis results of significant associations for "Tools"

Theme/Subtheme	Factor	n (%) or Mean age ± SD	P-value
Professionalism	Doctor	127 (48.8)	0.028 [1]
	Nurse	38 (36.2)	
Lifestyle advice	Doctor	0 (0.0)	0.000 [2]
	Nurse	8 (7.6)	
Working with colleagues	Doctor	64 (23.7)	0.049 [1]
	Nurse	17 (14.8)	
Continuity of care	Urban	0 (0.0)	0.036 [2]
	Rural	3 (2.7)	
Competencies in communication	Only primary care team	59 (21.6)	0.037 [1]
	Primary Care Emergency Service+out-of-hospital emergencies+Both of them+Others	8 (10.8)	
Competencies in information technology	Only primary care team	24 (8.5)	0.011 [1]
	Primary Care Emergency Service+out-of-hospital emergencies+Both of them+Others	15 (18.3)	
Delegation and coordination of care	Age (years)	47.4±9.4 vs 45.8±12.2 [4]	0.453 [3]
Time management	Age (years)	52.3±10.1 vs 45.5±12.0 [4]	0.004 [3]

[1] Chi-square test; [2] Fisher's exact test; [3] Student's t-test; [4] Mean age ±SD of respondents reporting tools versus those not reporting them

Additional file 18:

TABLE AF18. Multivariable logistic regression models (significant predictors only)

Outcome	Predictor	Adjusted OR (95% CI)	p-value
**Training needs**			
Medicines management	Female gender (vs male)	0.58 (0.34–0.99)	0.044
	Profession (nurse vs physician)	0.46 (0.28–0.76)	0.002
	Work setting (urban vs rural)	0.60 (0.36–0.99)	0.046
Joint decision-making and goal setting	MULTIPAP participation (yes vs no)	4.80 (1.52–15.13)	0.007
Information-technology skills	Urban setting (vs rural)	3.52 (1.02–12.17)	0.047
Lifestyle advice	Profession (nurse vs physician)	3.35 (1.30–8.64)	0.012
	Resident status (vs non-resident)	3.84 (1.04–14.17)	0.043
**Difficulties**			
Communication	MULTIPAP participation (yes vs no)	2.66 (1.14–6.17)	0.023
Delegation and coordination of care	MULTIPAP participation (yes vs no)	2.97 (1.33–6.63)	0.008
Time management	Profession (nurse vs physician)	0.42 (0.25–0.71)	0.001
Complex care pathways	Profession (nurse vs physician)	1.98 (1.04–3.75)	0.037
Community health	Profession (nurse vs physician)	3.05 (1.20–7.73)	0.019
	Work setting (urban vs rural)	0.34 (0.13–0.86)	0.023
Clinical skills	Work setting (urban vs rural)	0.51 (0.32–0.81)	0.004
**Training priorities**			
Medicines management	Profession (nurse vs physician)	0.26 (0.14–0.50)	<0.001
Communication	MULTIPAP participation (yes vs no)	3.20 (1.26–8.14)	0.015
	Profession (nurse vs physician)	2.25 (1.13–4.48)	0.022
Complex care pathways	Female gender (vs male)	0.57 (0.33–0.99)	0.047
	Resident status (vs non-resident)	2.86 (1.02–8.05)	0.046
	Work setting (urban vs rural)	0.49 (0.29–0.84)	0.009

Only predictors with $p < 0.05$ are displayed. Entries show Adjusted Odds Ratio (aOR) with 95% Confidence Interval (CI) and p-value. Goodness-of-fit (Hosmer–Lemeshow) was acceptable in all multivariable logistic models (all $p > 0.05$)
